# SICT: automated detection and supervised inspection of fast Ca^2+^ transients

**DOI:** 10.1038/s41598-018-33847-4

**Published:** 2018-10-19

**Authors:** Roberta Mancini, Tobias van der Bijl, Quentin Bourgeois-Jaarsma, Rizky Lasabuda, Alexander J. Groffen

**Affiliations:** 1 Department of Functional Genomics, Faculty of Science, Center for Neurogenomics and Cognitive Research, Vrije Universiteit, De Boelelaan 1085, 1081HV Amsterdam, The Netherlands; 2 Department of Clinical Genetics, Center for Neurogenomics and Cognitive Research, VU Medical Center, De Boelelaan 1085, 1081HV Amsterdam, The Netherlands

## Abstract

Recent advances in live Ca^2+^ imaging with increasing spatial and temporal resolution offer unprecedented opportunities, but also generate an unmet need for data processing. Here we developed SICT, a MATLAB program that automatically identifies rapid Ca^2+^ rises in time-lapse movies with low signal-to-noise ratios, using fluorescent indicators. A graphical user interface allows visual inspection of automatically detected events, reducing manual labour to less than 10% while maintaining quality control. The detection performance was tested using synthetic data with various signal-to-noise ratios. The event inspection phase was evaluated by four human observers. Reliability of the method was demonstrated in a direct comparison between manual and SICT-aided analysis. As a test case in cultured neurons, SICT detected an increase in frequency and duration of spontaneous Ca^2+^ transients in the presence of caffeine. This new method speeds up the analysis of elementary Ca^2+^ transients.

## Introduction

Ca^2+^ is a ubiquitous, conserved second messenger that regulates a range of cellular processes in all kingdoms of life^1–3^. In excitable cells, such as muscle cells and neurons, Ca^2+^ triggers contraction and neurotransmission, respectively^4,5^. Fluorescence microscopy allows the spatial and temporal measurement of intracellular Ca^2+^ dynamics in living cells^6,7^. Research questions linked to Ca^2+^ pathways are widespread in science and require different tissues or cell types, Ca^2+^ reporters and imaging hardware.

## Neuronal Ca^2+^ transients

While classical neurotransmission is long known to be governed by action potential (AP)-evoked Ca^2+^ transients, there is growing interest in various other types of Ca^2+^ signals and their putative role in synaptic plasticity and spontaneous neurotransmission^8–11^. In contrast to AP-evoked transients, smaller amplitude signals - referred to as Ca^2+^ sparks, Ca^2+^ puffs, Ca^2+^ syntillas or spontaneous Ca^2+^ transients (SCTs) - occur locally in restricted subcellular compartments and are relatively difficult to detect. Ca^2+^ sparks/sparklets are local Ca^2+^ transients that are commonly observed in skeletal and cardiac muscle cells. By definition, they originate from single Ca^2+^ release units in the sarco- or endoplasmic reticulum where each unit represents a cluster of Ca^2+^ release channels such as ryanodine receptors (RyRs), inositol 1,4,5-trisphosphate receptors (IP_3_Rs), or a combination of both^12,13^. These receptors are also expressed in nervous tissues and sparks have been observed in hippocampal and dorsal root neurons^14,15^. So-called Ca^2+^ puffs are thought to represent cytosolic Ca^2+^ rises released from intracellular Ca^2+^ stores, again with a contribution of IP_3_Rs^16^. Ca^2+^ syntillas are defined as Ca^2+^ sparks in presynaptic terminals, for instance in hypothalamic neurons, where they occur spontaneously in the absence of APs and extracellular Ca^2+^ by a RyR-dependent mechanism^17^. Finally, spontaneous Ca^2+^ transients (SCTs) occur in the absence of AP and involve RyRs. They were observed in cerebellar Purkinje cells^18^ and hippocampal neurons^8,11^. The recurrent role of intracellular Ca^2+^ channels in Ca^2+^ sparks, puffs, syntillas and SCTs suggests a shared mechanism for these types of events. Alternatively, spontaneous Ca^2+^ transients can also originate from the spontaneous opening of voltage-dependent Ca^2+^ channels at resting membrane potential, as demonstrated in cultured hippocampal neurons^10^. For simplicity, we will here collectively refer to all types of events as SCTs. The local and short-lived nature of SCTs (duration in the range of 40–260 ms depending on the reporter used), together with their low amplitude (ΔF/F_0_ typically in the 0.3–1 range) and frequency (0.005–0.7 Hz) make the detection of these events challenging^8,14,15,18^.

Cytosolic Ca^2+^ concentrations can be monitored with both chemical and genetically-encoded Ca^2+^ indicators (GECI), each with their own advantages and limitations^19–22^. In the latter class, the GCaMP family^23^ has been improved by altering its Ca^2+^ affinity, brightness, and subcellular localization^24–26^ to yield variants with high Ca^2+^ affinity and fast kinetics, including GCaMP6f^27^. Another key factor for the detection of intracellular Ca^2+^ fluctuations is related to imaging hardware. For optimal temporal resolution and light sensitivity, high-quality oil-immersion objectives offer high numerical apertures. Vacuum-cooled electron multiplying CCD (EM-CCD) cameras detect photons with quantum efficiencies above 90% while maintaining reasonable noise levels. Inevitably, there is a trade-off between the image capture time (accumulating enough photons to retrieve a good signal-to-noise ratio) and time resolution (the framerate needed to capture fast biological events)^19^.

## Image processing

High quality real-time Ca^2+^ imaging yields valuable datasets, but the large file sizes and the short duration of transient signals in noisy image data pose a challenge in data processing and storage. Considering the need to compare conditions in many cells per groups, manual processing of Ca^2+^ imaging data is laborious and sensitive to human bias by gradual changes in decision-making. Many good programs have been developed to detect changes in Ca^2+^ imaging data. Most are open source and available for widely used platforms as ImageJ^28,29^, MATLAB^30–39^, Python^38,40^ and R^29^ or can be run as a desktop application^41^. Many excellent tools, such as SeNeCA^30^,NeuroCa^31^ and FluoroSNNAP^32^, were designed for the optical analysis of neuronal circuits or high-throughput analysis of cell cultures, typically reporting Ca^2+^-based activity patterns of single or multiple neuronal cell bodies detected *in vivo*, *ex vivo* or *in vitro*^30–32,36,37,39,41^. Other tools like PeakCaller^35^ and SamuROI^40^ analyse Ca^2+^ signals in predefined regions (ROIs), offering accurate detection of biological signals in noisy data on the macro- to micro-scale, as small as dendritic spines. Automated detection of micro-scale Ca^2+^ signals in spatiotemporal (x,y,t) datasets has been achieved with xySpark in cardiomyocytes loaded with Fluo-4-AM (short duration with halftimes in the 10–50 ms range; spatial halfwidth of 1–5 µm)^28^ and with CaSCaDe in astroglia expressing GCaMP3 (mean duration of 9 s, size range 6–22 µm)^33^. For our purpose of studying spontaneous synaptic Ca^2+^ signals however, we were prompted to develop an algorithm combining all of the following features: (a) the ability to detect low-amplitude signals in noisy data with a small type II error (i.e. with a good detection rate of biological events); (b) analysing full images without user-defined ROIs; (c) the ability to process large movies (typically 10 GB in size); (d) analysing events in micro-scale subcellular compartments rather than somatic oscillations or patterns in neuronal ensembles; (e) the ability to visualize spatiotemporal signals and categorize the resulting events; and (f) tools to report descriptive parameters of the detected events including amplitude, frequency and kinetics.

In general, automated detection methods employ four steps. First, data are filtered to reduce noise. The standard deviation of a Gaussian filter is typically implemented as a user controlled setting and can be applied to the spatial or temporal dimension of a signal, or both. Second, a threshold is set based on data characteristics, such as the mean and the standard deviation of the spatial and/or temporal dimension of the data. Data are then divided into super threshold and sub threshold pixels, where super threshold pixels are considered to contain relevant biological signal whereas sub-threshold pixels are excluded from further analysis. Third, super-threshold pixels that are directly adjacent in the spatial or temporal dimension are grouped into individual segments, which can be further analysed as distinct units of the data (‘regions of interest’). A widely used method for grouping is connected-component analysis. Other strategies such as spatio-temporal independent component analysis (ICA)^32,36^, non-negative matrix factorization (NMF)^37^ and constrained non-negative matrix factorization and deconvolution^38^ exist but were not evaluated in our study.

Here we report SICT, an open source program that automatically detects low amplitude SCTs in noisy time-lapse imaging data. SICT allows to visually inspect each Ca^2+^ event and classify events for further analysis. The validity and sensitivity of the methods were confirmed using both simulated and experimental data.

## Results

### Automated detection of SCTs

**Figure 1:**
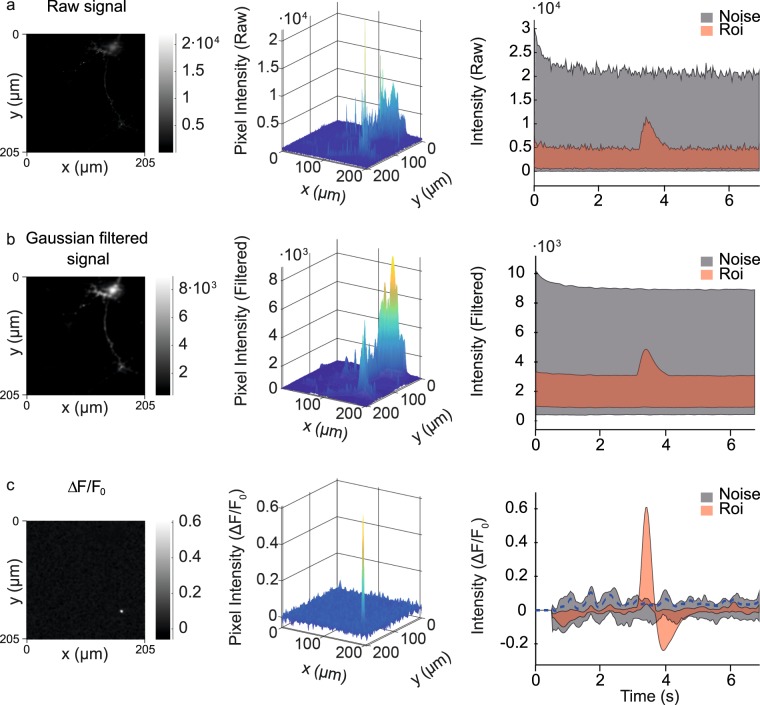
Spatiotemporal effects of filtering. As an example, the Ca^2+^ indicator GCaMP6f was expressed in hippocampal neurons and recorded by live epifluorescence microscopy. Three stages of signal processing are illustrated from top to bottom. For each stage, a single 2D image is depicted as grayscale image (left), as surface graph (middle) or as quantitated signal over time (right). In the right graph, the red area indicates the maximal and minimal signal of a 2D ROI consisting of 372 pixels in a region where a Ca^2+^ transient occurred. The grey area indicates the maximal and minimal signal in a background region (defined as all other 261772 pixels). A Ca^2+^ transient peak occurred at (x, y, t) = (166 µm, 157 µm, 3.48 s). (**a**) The raw signal demonstrates the difficulty of identifying Ca^2+^ events in raw data. (**b**) A 3D Gaussian filter with a standard deviation of 3 and 2 pixels respectively in the spatial and temporal dimension was applied to remove high-frequency spatial and temporal noise. (**c**) The ‘ΔF/F_0_’ transformation highlights pixels that have increased intensity values compared to the previous frames (left, middle). The ΔF/F_0_ signal exceeds the background, allowing peak detection in noisy data (right). The threshold used for automated event detection is defined as the median pixel intensity of each frame plus three times the interquartile range (blue dashed line).

Cultured neurons expressing the fluorescent Ca^2+^ indicator GCaMP6f were studied by time-lapse imaging at ≈29 Hz. Exploiting repeated measurements in the time dimension, a 3D Gaussian filter effectively reduced noise as illustrated in Fig. 1a,b. To optimize the detection of sudden increases of Ca^2+^, occurring within less than 5 frames (0.17 s in our experiments), ΔF/F_0_ values were calculated using a moving average for F_0_ (see methods for details). Note that this definition of F_0_ is unsuitable to detect slow fluctuations. A threshold was set at the median plus 3 times the interquartile range for each frame (Fig. 1c, right panel). All pixels exceeding the threshold were grouped together with neighbours in space or time, resulting in 3D ROIs containing various numbers of pixels. Each ROI thus corresponds to a putative Ca^2+^ event.

To reduce false positive detections, the ROIs were subsequently filtered based on two criteria. First, ROIs consisting of only a single pixel were excluded because these events typically originate from ‘hot pixel noise’ generated in the EM-CCD camera, and are inconsistent with the instrument’s point spread function. Second, ROIs occurring outside the neuronal cell structure can optionally be excluded. For this purpose, an inclusion mask was defined as all image regions with above-average pixel intensities (Supplementary Fig. 1). The latter criterion reduced the number of ROIs twofold. In 14 cells analysed, 98,4% of all SCTs (interpreted as true events after visual inspection) occurred within the neuronal inclusion mask, suggesting that this filter has a minor effect on detection sensitivity (Supplementary Fig. 1). All ROIs meeting the inclusion criteria were saved for later visual inspection and downstream analysis together with their properties (see Supplementary Table 1).

### Performance of automated detection in noisy data

**Figure 2:**
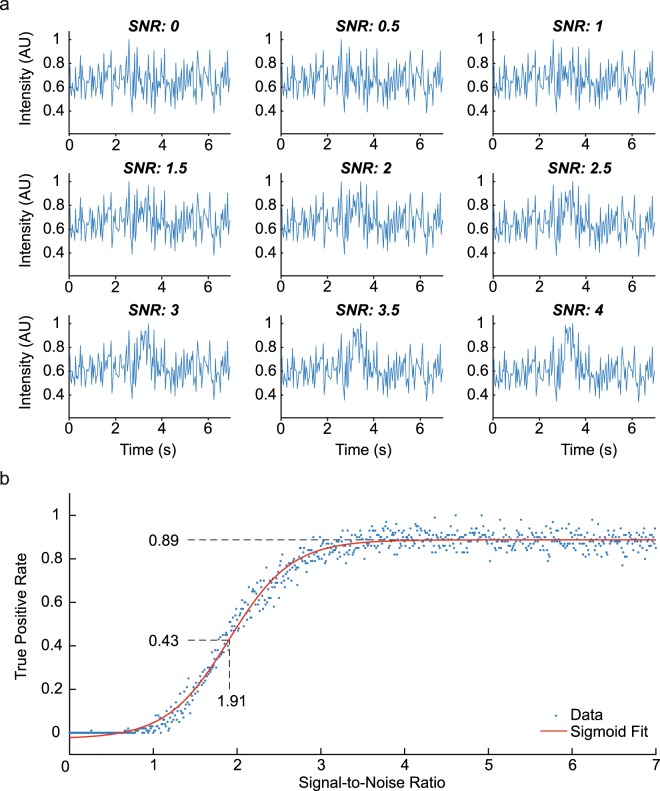
Program performance. True positive detection rate as a function of SNR in simulated noisy datasets. (**a**) Example traces of simulated raw data for a range of SNR. A pixel was chosen randomly and simulated with a SNR from 0 to 4 in steps of 0.5, using the same simulated background noise and example event. (**b**) The performance of automated detection was measured as the fraction of accurately detected events in simulated datasets with different SNRs. Each blue dot represents a separate test. The red line indicates a sigmoid fit of the aggregate data. Half-maximal performance was observed at SNR 1.91 with a true positive rate of 0.43. Optimal performance (99% of the maximum rate of 0.88) was reached at SNR 3.64 and higher.

To test the performance of SICT in the presence of increasing noise levels, simulated datasets were produced at different SNRs by mixing synthetic Ca^2+^ signals with realistic background noise. Figure 2a illustrates simulated events with identical background noise in a range of SNRs. At maximum performance, 89% of the generated events were correctly detected. Of this maximum performance, 99% was reached at a SNR of 3.64. Half/maximal performance was reached at 1.91 SNR (Fig. 2b).

### Assisted inspection

To allow visual inspection of the ROIs generated by automated detection, we developed a graphical user interface which is explained in detail in the Supplemental Manual. During the manual selection, ROIs can be sorted for descending quality scores which helps to identify true events (see Supplementary Fig. 6). Manual selection further allows to categorize different classes of Ca^2+^ signalling events. The graphical interface shows each event, both after signal processing and as raw data. Relevant sections of the time-lapse movie can be played using shortcut keys, allowing to distinguish putative biological events from various potential artefacts like moving structures, focus drift, hot pixel noise or any other possible sources of false event detection. We defined an event as true if the identified peak is consistent with the appearance of Ca^2+^ fluctuations in living cells.

After inspection, ROIs scored as true events were analysed for their properties such as the event frequency, amplitude, rise time, decay time, and FWHM (full width at half maximum; Supplemental Manual, step 3). Common properties are shown in Supplementary Fig. 2. Optionally, the user can label events in 9 classes based on their kinetics. Different types of Ca^2+^ kinetics were observed in our experiments, summarized in Supplementary Fig. 3 and Supplementary Table 2. In 14 cells analysed, fast isolated Ca^2+^ transients (‘FastSingle’) represented the most abundant class of events (59.69%). Other events appeared as a combination of fast and slow Ca^2+^ rises (‘SlowFastComplex’; 18.86%), included multiple fast Ca^2+^ transients (‘FastComplex’; 9.59%) or were classified as slow isolated Ca^2+^ transients (‘SlowSingle’; 5.95%). Other event types had minor contributions (Supplementary Fig. 3).

### Human observer comparison

To evaluate the effect of differences between individual users during event inspection, four human observers were instructed to select all fast-isolated Ca^2+^ transients based on experience and a list of guidelines: the Ca^2+^ event should be isolated (and therefore not surrounded by other Ca^2+^ events), spatially localized in the neuronal structure and short-lived (in the order of ms). The same four recordings were processed by each observer. Before inspection, ROIs were sorted from high to low amplitude. Observers were instructed to continue searching for Ca^2+^ transients until no single transients were detected in the last examined 320 events.

**Figure 3:**
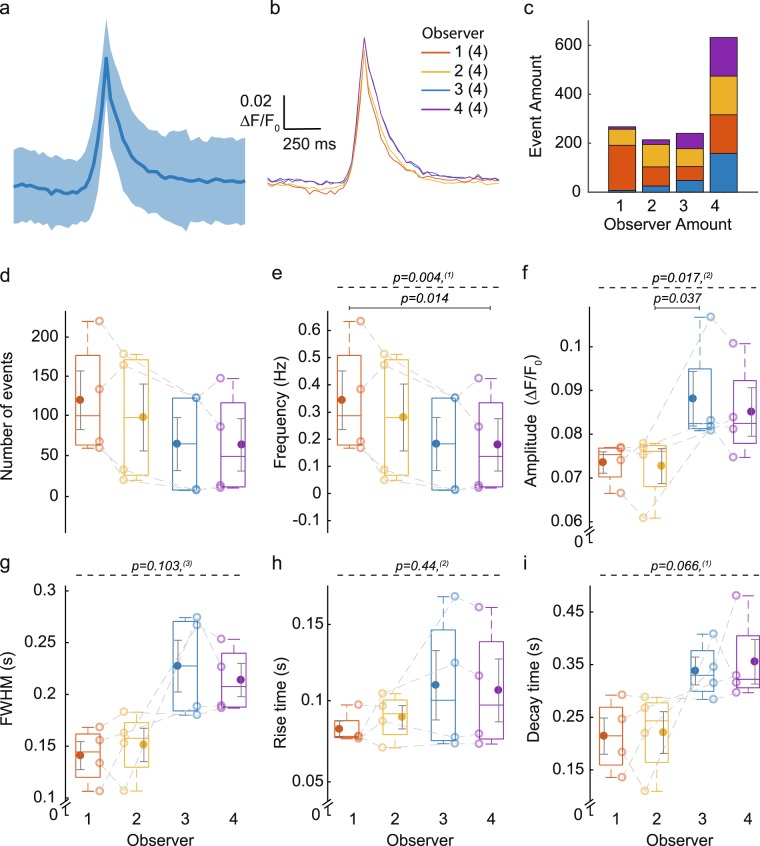
User-dependence of Ca^2+^ event inspection. Four human observers inspected ROIs from the same dataset and selected ‘FastSingle’ Ca^2+^ events (Supplementary Fig. 3). (**a**) Average ΔF/F_0_ trace ± SD of 612 unique events selected by one or more observers. (**b**–**i**) Each colour (orange, yellow, blue and purple) marks a different observer. (**b**) Average ΔF/F_0_ trace of all events selected by each observer. (**c**) Number of events selected by one or multiple (1–4) observers. (**d**–**i**) Comparison of event parameters after scoring by different observers is shown in box plots. Open circles show mean value per cell; closed circle represent the mean ± SEM of n = 4 cells, N = 1 independent experiment. Statistical analysis was performed with ^(1)^one-way repeated measures ANOVA; ^(2)^Friedman test, ^(3)^one-way repeated measures ANOVA with Greenhouse-Geisser correction (dashed line); pairwise comparison (line). (**d**) Number of events. (**e**) Frequency. (**f**) Amplitude. (**g**) FWHM. (**h**) Rise time. (**i**) Decay time.

Between the four human observers, of all 612 unique events scored by at least one observer (average trace ± SD shown in Fig. 3a), 158 events were scored by each of the four observers. One observer selected over 250 unique events that were not scored by other users (Fig. 3c). The average trace per observer did not show meaningful differences (Fig. 3b). We also compared the number of events, frequency, amplitude, FWHM, rise time and decay time (Fig. 3d–i). Collectively, the results show that the frequency of Ca^2+^ transients scored by different observers varies significantly. A similar difference was found for the amplitude, although with a small effect size. It is thus advisable that different experimental groups are analysed by the same person.

### Manual versus assisted image processing

**Figure 4:**
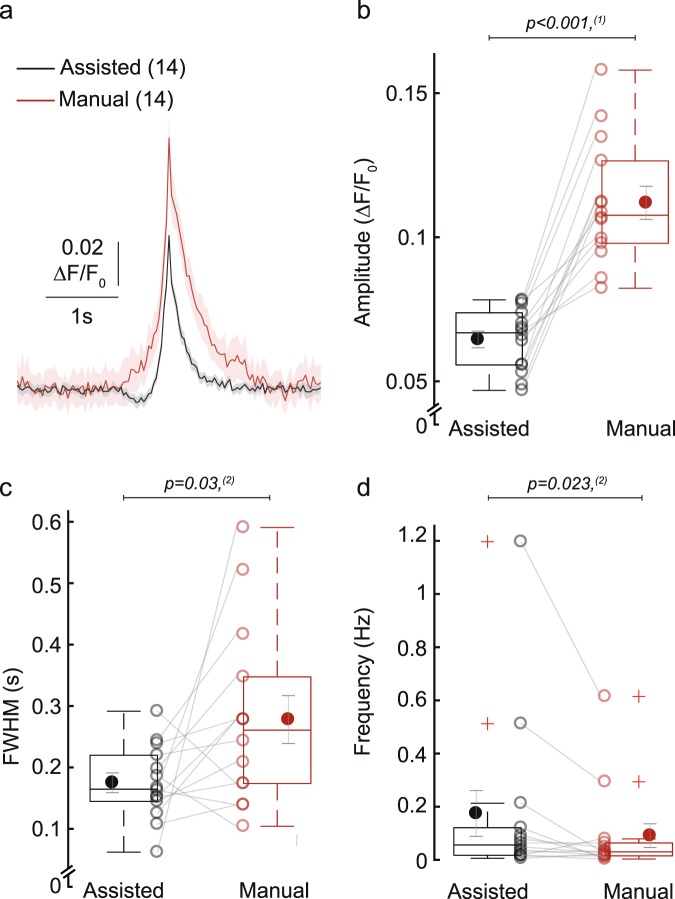
Comparison of manual and assisted image processing. (**a**) Average ΔF/F_0_ trace ± SEM of all detected Ca^2+^ peaks after manual (red) or assisted image processing (black). Traces were first averaged to calculate the mean trace per cell, and then averaged to calculate the mean ± SEM from n = 14 cells, N = 1 independent experiment. (**b**–**e**) Box plots show Ca^2+^ transient parameters resulting from assisted and manual image processing. Open circles represent averaged value per cell; closed circles show mean ± SEM from 14 cells, N = 1 independent experiment. Statistical significance was tested with ^(1)^paired samples t-test; ^(2)^Wilcoxon signed-rank test. (**b**) Amplitude. (**c**) FWHM. (**d**) Frequency.

To validate the assisted image processing method, we compared the results from manual and assisted processing obtained from the same 14 time-lapse movies (Fig. 4). A key difference is that in manual processing, the ROIs are defined *a priori* by the user and do not change over time. In contrast, the assisted method includes all pixels with detectable fluorescence signals, creating a ROI that can change over time. High-intensity Ca^2+^ events produce more pixels with detectable signals, yielding larger ROI sizes from which the ΔF/F_0_ signal is averaged. Thus, the amplitude of a Ca^2+^ event is not directly proportional to the fluorescence intensity of the raw signal.

In line with this idea, the assisted procedure reported significantly lower peak ΔF/F_0_ amplitudes of 0.064 compared to 0.112 after manual detection (p < 0.001; Fig. 4a,b and Supplementary Fig. 4). The number of detected events was higher after assisted detection (frequency of 0.174 Hz compared to 0.091 Hz; Fig. 4d,e) but this difference was not significant. Furthermore, the peak duration tended to be longer after assisted detection (FWHM of 0.278 s compared to 0.175 s; Fig. 4c and Supplementary Fig. 4).

**Figure 5:**
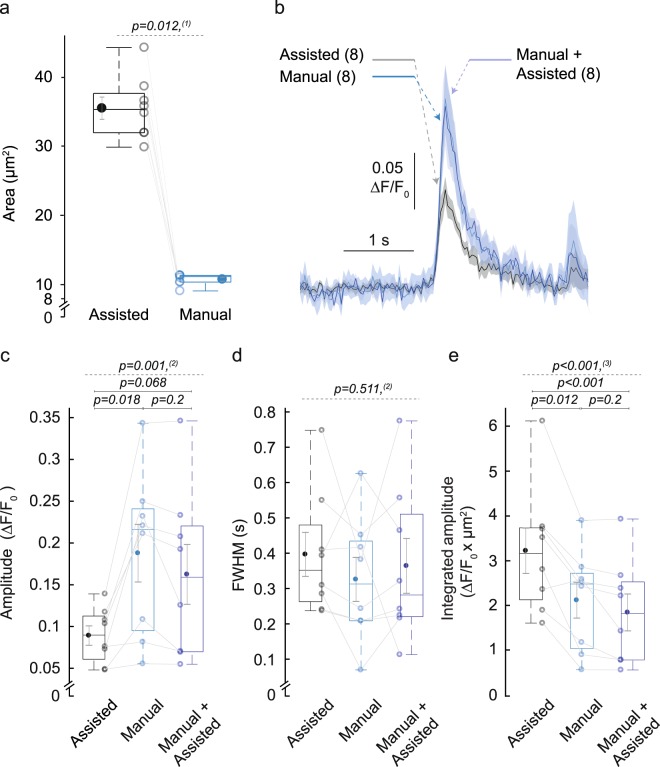
Effect of ROI definition on Ca^2+^ event parameter measurement. (**a**) Automatically identified ROIs (black) had a bigger 2D area than manually drawn ROIs (dark blue). (**b**–**e**) Each parameter was calculated using the fully assisted (black) and the manual method (dark blue), or a combination where the ROIs were manually defined and further processed by the assisted method (light blue). (**b**) Average ΔF/F_0_ trace ± SEM of 8 Ca^2+^ transients of 1 cell. (**c**–**e**) Box plots show Ca^2+^ event parameters. (**c**) Amplitude. (**d**) FWHM. (**e**) Integrated amplitude. Statistical significance was assessed with ^(1)^Wilcoxon signed-rank test, ^(2)^one-way repeated measures ANOVA or ^(3)^one-way repeated measures ANOVA with Greenhouse-Geisser correction (dashed line); pairwise comparison (line; n = 8 Ca^2+^ events, N = 1 cell).

To verify if the ROI area was an important determinant of the reported SCT amplitudes, the ROI areas in the x, y dimension were compared for the same SCTs detected by the manual vs. the assisted method. The mean area of automatically identified ROIs was more than three-fold bigger (35.5 ± 1.6 µm^2^ compared to 10.8 ± 0.3 µm^2^; n = 8 events from 1 cell, Fig. 5a). To further test the effect of the different ROI definition on event amplitude and duration (Fig. 5b–d), we processed the same 8 Ca^2+^ transients in three different ways: (1) with ROIs generated by assisted processing as before; (2) with ROIs that were manually drawn as before; and (3) with ROIs identical to the manual method, but further processed by the assisted method. As expected, the lower amplitude of automatically processed ROIs (0.089 ± 0.012 ΔF/F_0_ for group 1 and 0.188 ± 0.035 for group 2; p = 0.018) was restored to a similar amplitude when the ROIs were made identical (group 3, 0.162 ± 0.036 ΔF/F_0_; p = 0.068 compared to group 1). Differences in the reported peak duration were not statistically significant between the three groups (Fig. 5d). To confirm the effect of the ROI area in the amplitude output, we calculated the integrated amplitude (area times amplitude), which shows a significantly higher value for the assisted processing (3.21 ± 0.51 ΔF/F_0_ × µm^2^ for group 1 and 2.08 ± 0.41 for group 2; p = 0.012) and even higher differences when the ROIs were made identical (group 3; 1.81 ± 0.42 ΔF/F_0_ × µm^2^; p < 0.001; Fig. 5e). By including all pixels with above-threshold signal, the automated ROI definition was more suitable for Ca^2+^ event detection in noisy data than the manual ROI definition. Thus, while the larger ROI size precludes a direct comparison of ΔF/F_0_ amplitudes, an optimal measure of peak intensity was obtained by integrating ΔF/F_0_ signal over ROI size.

### Caffeine effect on Ca^2+^ transients

To address if the assisted program is able to detect biological differences, we tested the effect of caffeine on SCT properties. Caffeine sensitizes RyRs, which in turn triggers Ca^2+^ release from intracellular stores^42^. The effect of caffeine on intracellular Ca^2+^ stores has been studied in many cell types, such as smooth and skeletal muscle cells, chromaffin cells, PC12 cells and different types of neurons. Generally, caffeine application increases intracellular Ca^2+^ concentration in a cell type- and concentration-dependent manner^43–46^. The effect of caffeine on SCTs is well studied in excitable cells such as smooth muscle cells, in which caffeine increases the frequency of SCTs^12^. Caffeine also increases the frequency of SCTs in magnocellular hypothalamic neurons^17^ and superior cervical ganglion neurons^47^.

**Figure 6:**
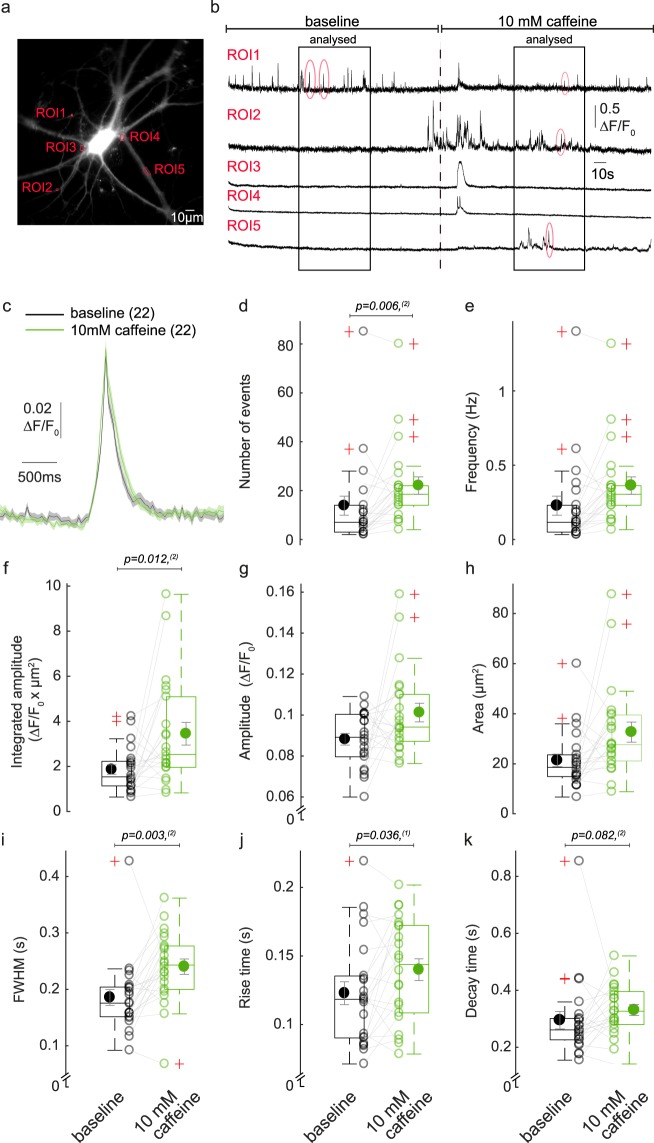
Caffeine affects the frequency and duration of SCTs. (**a**) Representative image of a neuron labelled with GCaMP6f. Red squares indicate five ROIs for which the ΔF/F_0_ intensity over time is shown in **b**. (**b**) Each neuron was perfused for three minutes in 1 μM TTX followed by three minutes in 1 μM TTX and 10 mM caffeine. Only the second minute of each perfusion was used to analyse the properties of SCTs (red circles). (**c**–**k**) SCT event parameters in absence (black) and presence (green) of 10 mM caffeine. (**c**) Average ΔF/F_0_ trace ± SEM of all averaged SCTs per cell. (**d**–**m**) Box plots show averaged SCTs parameter values per cell (open circles) and their overall mean (closed circles). Statistical significance was tested using ^(1)^paired samples t-test; ^(2)^Wilcoxon signed-rank test (n = 22 cells, N = 3 independent experiments). (**d**) Number of SCTs. (**e**) Frequency. (**f**) Integrated amplitude. (**g**) Amplitude. (**h**) ROIs area 2D. (**i**) FWHM. (**j**) Rise time. (**k**) Decay time.

To investigate the effect of caffeine on SCTs in our hippocampal neurons, time-lapse images were recorded first for 3 min in the absence and then for 3 min in the presence of 10 mM caffeine. AP propagation in the neurons was blocked by TTX throughout the experiment. Ca^2+^ transients were analysed for the second minute of each condition to exclude image drifting artefacts and long-lasting events that were mostly noticed in the first minute after the application of caffeine (Fig. 6b). The number of SCTs was significantly increased by caffeine (from 13.8 ± 3.9 to 22 ± 3.6 events, corresponding to frequencies of 0.23 and 0.36 Hz respectively; p = 0.006, n = 22 cells, N = 3 independent experiments). The peak duration also increased from 185 ± 14 to 240 ± 36 ms (p = 0.003). A trend towards an increase was observed for the integrated peak intensity, from 1.89 ± 0.21 to 3.47 ± 0.5 ΔF/F_0_ × µm^2^ (p = 0.012; accepted α significance <0.01 for multiple parameters testing). The ROI area in presence of caffeine was 1.53-fold bigger than in its absence (Fig. 6h). In summary, these results show that SICT successfully identifies biological differences. Furthermore, we confirm that caffeine evokes complex, long-lasting Ca^2+^ events especially immediately after the drug application, and show that the frequency and FWHM of SCTs are increased one minute after caffeine application.

## Discussion

In the last years, several programs have been developed for (semi)-automatic Ca^2+^ signalling analysis in different software environments. For the purpose of our study, we aimed to develop a program that speeds up the detection of small-amplitude Ca^2+^ elevations in noisy data.

Events detected by the SICT algorithm still require manual curation. This was a deliberate decision and a key feature which distinguishes SICT from other excellent tools^28–35,40,41^, such as FluoroSNNAP^32^, xySpark^28^ and NeuroCa^31^. All are able to detect SCTs. When adjusting parameters to strictly filter out true biological signals, we found that such filtering inevitably reduces the detection sensitivity, leading to false negatives. In addition, we did not want to make strict assumptions regarding the signal waveforms to be detected. Thus, we aimed to make the manual curation very fast by building a convenient graphical interface. Importantly, the automatically detected events are sorted for their likeliness to represent a biological signal. Various sorting methods can be chosen as explained in the section ‘ROI sorting methods’ of the SICT user manual (see Supplemental information). Events with the highest scores are shown first, typically representing convincing biological events, whereas later in the list, true events will be accompanied by more and more false positive hits. At some point, when biological signals are scarce and noise becomes predominant, the user can decide to stop the manual curation process. This decision can also be seen as a ‘filtering threshold’, but it is not a blind choice because the user is aware of the signal and noise patterns specific for the experiment.

The assisted method was validated by testing its detection sensitivity using experimental data and simulated experiments with various SNRs. The program performance reached a maximum true positive rate (TPR) of 0.89, thus leaving 11% of true events undetected even at high SNRs. As a possible cause, local pixel noise may contribute to mislocation of peak signals in the x, y or time dimension. In another study, localization errors between 0 and 1 μm were reported^48^ whereas our criterion for effective detection was set to 0.4 μm. Half-maximal performance with our program required SNRs of 1.91. Other published programs have claimed high performance (>98%) with SNRs as low as 0.2 albeit calculated differently^49^. However, the method in that study used predefined ROIs as input, and furthermore used manually selected example events to train a classifier. We avoided such conditions to be able to analyse large numbers of time-lapse movies. Furthermore, as illustrated by the example events in Fig. 2a, it may be acceptable to exclude events at SNRs below 1.5. A balance between true and false positive detection rates is an inherent dilemma in all Ca^2+^ imaging experiments and should be reconsidered for each individual experiment. This dilemma underlines the value of visual event inspection to aid data interpretation.

Compared to manual processing, the SICT-assisted method readily detected Ca^2+^ elevations with at least a similar sensitivity. SICT uses dynamic ROIs (i.e. changing over time) which are automatically generated instead of being user-defined. Higher Ca^2+^ signals yield larger ROIs. Therefore, the best measure of peak intensity is obtained by integrating the ΔF/F_0_ signal over ROI size. In contrast, ΔF/F_0_ amplitudes without integration should not be used as a direct measure of Ca^2+^ signal amplitude. For the same reason, the SICT method typically reports lower ΔF/F_0_ amplitudes (0.05–0.1) than manual methods (0.3–1)^8,14,15,18^. These lower ΔF/F_0_ amplitudes do not reflect lower Ca^2+^ peak concentrations but are a consequence of calculating the ΔF/F_0_ from a larger ROI, which helps to average out noise and improves the sensitivity of detection.

The SICT code is open source, allowing versatile downstream data processing in MATLAB. Both raw and analysed data are stored in a structure which allows easy data retrieval and event inspection. Overall, using the assisted processing and visual event inspection we could analyse 10 times more recordings compared to the manual method in the same amount of time. As may be expected, visual inspection by different users was a significant source of variation in the analysis, mostly affecting the event frequency. To avoid systematic differences, event inspection should be performed by the same user for all experimental groups.

As a test case, our program readily detected the expected increase in the frequency and duration of SCTs in paired recordings from hippocampal neurons after caffeine application. At the same caffeine concentration, Ca^2+^ syntillas in chromaffin cells previously showed a 1.73-fold frequency increase, without effect on the amplitude^50^. Nerve terminals stimulated with 20 mM caffeine exhibited a 2-fold frequency increase while the amplitude increased 1.18 fold^17^. Other studies on hippocampal neurons, which focused on SCTs, generally altered the SCTs using 30 μM ryanodine, which reduced the frequency of SCTs by blocking RyRs^11^. Finally, Llano and colleagues assessed SCTs in Purkinje cells after application of an agonistic concentration of ryanodine (5–10 μM), which induced a 3.8-fold frequency increase^18^. Our results thus agree with previous reports and demonstrate that SICT is well applicable to detect changes in SCT frequencies.

In conclusion, SICT-assisted image processing can be widely applied to investigate Ca^2+^ transients or other fast local fluorescence increases in live cell imaging data, providing a new tool to accelerate the discovery of fast local signalling pathways in living systems.

## Methods

### Primary neuron culture

Animals were housed, bred and experimentally used according to institutional guidelines and Dutch and U.S. governmental laws. Prior approval was obtained from the animal ethical committee of the VU University and VU Medical Centre, named ‘Dier ethische commissie (DEC)’, approval license FGA 11-03. Hippocampal neurons were obtained from C57BL/6 mouse brain at embryonic day E18. Unless specified otherwise, all chemicals were from Invitrogen (The Netherlands). After removal of the meninges, the hippocampi were dissected in Hank’s balanced salt solution (HBSS; Sigma), which was buffered with 10 mM HEPES. Cells were dissociated with 0.25% trypsin for 20 min at 37 °C. After washing 5 times in HBSS, the cells were suspended in Neurobasal medium, supplemented with 2% B-27, 1.8% HEPES, 0.25% glutamax and 0.1% Pen-Strep. Cells were triturated by three passes through fire-polished Pasteur pipettes and counted using a Fuchs-Rosenthal chamber. High density continental cultures were created by seeding 25k neurons on a layer of 25k rat glia per well on 18 mm coverslips in 12-well plates or 50k neurons on 50k glia on 25 mm coverslips in 6-well plates. Glass coverslips were pretreated by washing in ethanol and sprayed with 0.1 mg/ml Poly-D-lysine, 0.2 mg/ml rat tail collagen (BD Biosciences) in 10.2 mM acetic acid. Cultures were kept in a humidified incubator at 37 °C and 5% CO_2_. After 8 days, half of the medium was replaced. Neurons were infected with lentiviral particles encoding GCaMP6f^27^ at 7 days *in vitro* (DIV) and analysed between DIV14-18.

### Ca^2+^ imaging

Ca^2+^ imaging was performed using a custom-built epifluorescence setup called FAINT (for Flash activation of Action potential-Independent NeuroTransmission). It uses an inverted microscope (IX73, Olympus) illuminated by a polychromeV monochromator (TILL Photonics) with a 150 W Xenon high stability lamp in combination with a suitable GFP filter set (GFP filterset 49002, Chroma). Coverslips were placed in the imaging chamber with artificial cerebrospinal fluid (ACSF) as extracellular solution containing (in mM): 140 NaCl, 2.4 KCl, 10 HEPES, 10 glucose, 4 CaCl_2_, 4 MgCl_2_ (pH 7.3, 300 mOsm). 1 μM of tetrodotoxin (TTX, Ascent) and 10 mM caffeine were added when specified. GCaMP6f was excited at a wavelength of 480 nm. Imaging was performed using a 40x-oil immersion objective (Olympus UAPON40xO340-2, NA 1.35) at RT (22–24 °C). Images were acquired by Live Acquisition software (LA; FEI) at a frequency of 28.77 Hz for 3–5 minutes using an Andor Ixon Ultra 897 electron-multiplying CCD (EM-CCD) camera with the following settings: vacuum cooling to −70 °C, EM gain of 200, pre-amplification of 2, 40% lamp intensity, 30 ms exposure. Images had a spatial resolution of 512 × 512 pixels each calibrated to cover 0.16 μm^2^ of the specimen. Intensity values were recorded in 16-bit unsigned integer format, producing roughly 10 gigabytes per time-lapse movie. Data were exported in ‘.raw’ format using the ‘Offline Analysis’ data streaming option in Live Acquisition software, together with its associated ‘.mtd’ file which contains all metadata of the experiment. To investigate the effect of caffeine on SCTs, the same cell was first perfused in presence of 1 µM TTX for 3 min, followed by 3 min in 1 μM TTX and 10 mM caffeine.

### Automatic event detection

Source code is accessible in the Supplemental Software. All computations were performed in MATLAB version 2016b^51^ unless specified otherwise. The automatic detection of ROIs is run by a graphical user interface (see Supplemental Manual, step 1). Data was imported as a 3D matrix (x, y, time) of unsigned 16-bit integer numbers indicating GCaMP6f fluorescence intensity. Instead of the often used 2D filter^48^ we used a 3D Gaussian filter (imgaussfilt3) to reduce high-frequency noise by weighted averaging each pixel with its neighbours, using a standard deviation (sigma) of 3 pixels for spatial, and 2 frames for temporal filtering. To calculate ΔF/F_0_ for each pixel, the baseline intensity (F_0_) was determined as a moving average of 15 to 5 time points prior to the current data point. The ΔF/F_0_ was then calculated as (F − F_0_)/F_0_. For an example of typical raw and calculated data values, see Supplementary Fig. 8. The threshold to select Ca^2+^ transients was calculated for each frame by taking the median plus three times the interquartile range. The interquartile range is an effective measure of noise levels because it is not affected by the occurrence of Ca^2+^ fluctuations or baseline drift.

The above-threshold data points were clustered into 3-dimensional ROIs using the bwconncomp function. The resulting ROIs (each a collection of voxels in the x,y,t dimension) represent putative Ca^2+^ elevations. Information for each ROI was saved to an output file for further analysis, including raw intensity values, the peak x, y and t coordinates and the weighted centroid (for a full list of properties see Supplementary Table 1).

### Assisted event inspection

Kinetic parameters for single events are calculated as detailed in the Supplementary Table 3. In short, these included the baseline and amplitude of the ΔF/F_0_ trace; the 10–90% rise time, 90-10% decay time and the full width at half maximum (FWHM). For kinetic parameters, time values were estimated beyond framerate resolution by linear interpolation. To report descriptive parameters of selected SCTs, the parameters were first calculated per event, averaged per cell and then averaged to get the overall mean.

### Manual image processing

For comparison, we also performed manual processing by opening the image stack in ImageJ (NIH). The first 500 frames (17s) were removed to allow removal of a bleaching component by subtraction of a mono-exponential curve fitted to the average frame intensity over time. ROIs were manually drawn, together fully covering visible neuronal structures in the image. Average pixel intensities of each ROI were calculated for each frame, graphed and visually inspected. The ΔF/F_0_ was calculated using a F_0_ value calculated as the average from the last 25 data points of the trace. Peak amplitude was calculated as maximum ΔF/F_0_ during the peak minus a baseline value (averaged value from the 10 data points of the same ΔF/F_0_ trace, 50 frames before the peak). The FWHM was calculated as the time of the closest data point between the half-maximal signal in the rising and decay phase of the peak. The frequency of events was calculated as the total number of events divided by the total imaging time in each experiment. The parameters were calculated per event, averaged per cell and then averaged to get the overall mean.

### Simulations of algorithm performance in noisy data

To assess the detection performance, a range of signal-to-noise ratios (SNRs) was tested using simulated data. Background noise was simulated by taking raw data frames and calculating mean and standard deviation. These values were used to generate a matrix with the same spatial dimensions. To reduce processing time, a decreased time dimension was used. Per simulation, 100 Ca^2+^ events at 10 Hz (on average 0.35 per imaging frame) were generated by adding values of 1 at random locations within a cell mask in a matrix of all zeros. The mask corresponded to all pixels that had, on average in the time dimension, a higher-than-average intensity compared to the whole field of view (likely corresponding to a fluorescently labelled cell). The events were then spread out in the spatial dimension using a 2D Gaussian filter with a standard deviation of one pixel in both the x and y direction. Subsequently, the signal was convoluted in the time dimension with a normalized example event, for which we used the Ca^2+^ signal from a neuron firing a single action potential. This example event was multiplied by the standard deviation of the corresponding pixel and multiplied with a SNR factor. Finally, the matrix that contained background noise was summated with the matrix that contained simulated events and saved in the same format as experimental data files.

This procedure was repeated with SNRs varying from 0.01 to 7 in 0.01 increments. The files were automatically processed by SICT and the output automatically analysed to determine how many events had been detected, using the criterium that SICT detected a ROI with a spatial accuracy of 1 pixel (0.4 µm) and a temporal accuracy of 10 frames (±348 ms).

### Data analysis

Statistical analysis was performed using SPSS v.23.0 (IBM Corp., Armonk, NY, USA). Data are reported as mean ± SEM, except when specified otherwise. Data were checked for normality using the Shapiro-Wilk or Kolmogorov-Smirnov test. When required, other assumptions were tested: homogeneity of the variance was assessed with Levene’s test; the sphericity assumption was tested with Mauchly’s test. For normally distributed data, a parametric test was run; otherwise a non-parametric test was performed. If the sphericity assumption was not met, the Greenhouse-Geisser correction was used to adjust the degrees of freedom. For two groups, a paired samples t-test or Wilcoxon signed-rank test was performed. In case of more than 2 groups, one-way repeated measures ANOVA or Friedman test was used.

Where necessary, the alpha significance threshold was adjusted for multiple testing. In case of more than 2 groups, a Bonferroni correction was used to adjust the p-value for pairwise comparison, keeping the alpha significance threshold at 0.05. The effect size was calculated for the paired samples t-test as ${\rm{r}}=\sqrt{{{\rm{t}}}^{2}/({{\rm{t}}}^{2}+{\rm{df}})}$, for Wilcoxon signed-rank test as ${\rm{r}}=|{\rm{Z}}/\surd {\rm{N}}|$, for one-way repeated measures ANOVA as ${\rm{r}}=\sqrt{{{\rm{\eta }}}^{2}}$ and, for Friedman test as ${\rm{r}}=\sqrt{{{\rm{\chi }}}^{2}/({{\rm{\chi }}}^{2}+{\rm{N}})}$. The effect size is reported for all p-values lower than 0.05. Statistical details for each figure are reported in Supplementary Table 4. In box plots, open circles represent individual measurements and filled circles depict mean ± SEM. The central line shows the median and statistical outliers are marked by red ‘plus’ symbols.

### Code availability statement

MATLAB code can be retrieved without restrictions from the VU Institutional Research Data Management system (Groffen, AJA, 2018, “Replication data for: SICT: automated detection and supervised inspection of fast Ca^2+^ transients”, https://hdl.handle.net/10411/UA6UZG, DataverseNL). An instruction is included in the Supplementary Information.

## Electronic supplementary material

Supplementary Information

## Data Availability

Replication data are deposited in the VU Institutional Research Data Management system (Groffen, AJA, 2018, “Replication data for: SICT: automated detection and supervised inspection of fast Ca^2+^ transients”, https://hdl.handle.net/10411/UA6UZG, DataverseNL).
