# Supplementary material for: SICT: automated detection and supervised inspection of fast Ca2+ transients
Source: Sci Rep. 2018 Oct 19;8:15523. doi: 10.1038/s41598-018-33847-4 (PMC6195629; doi:10.1038/s41598-018-33847-4)
Supplement: Supplementary file 1 — Supplementary Information [file 41598_2018_33847_MOESM1_ESM.pdf]

SICT: automated detection and supervised inspection of fast Ca^{2+} transients

Roberta Mancini[1], Tobias van der Bijl[1], Quentin Bourgeois-Jaarsma[1], Rizky Lasabuda[1] and Alexander J. Groffen[1,2,*]

[1]Department of Functional Genomics, Faculty of Science, Center for Neurogenomics and Cognitive Research, Vrije Universiteit, De Boelelaan 1085, 1081HV Amsterdam, The Netherlands.
[2]Department of Clinical Genetics, Center for Neurogenomics and Cognitive Research, VU Medical Center, De Boelelaan 1085, 1081HV Amsterdam, The Netherlands.
[*]Corresponding author, E-mail: a.j.a.groffen@vu.nl

SUPPLEMENTARY INFORMATION

Page 2 – 10: Supplementary figure S1 – S8

Page 11 – 23: SICT user manual (includes Supplementary Tables 1 – 3)

Page 24 – 26: Supplementary Table 4 (Details of statistical tests)

Supplementary Figure 1. Effect of a cell image mask on true positive event detection.

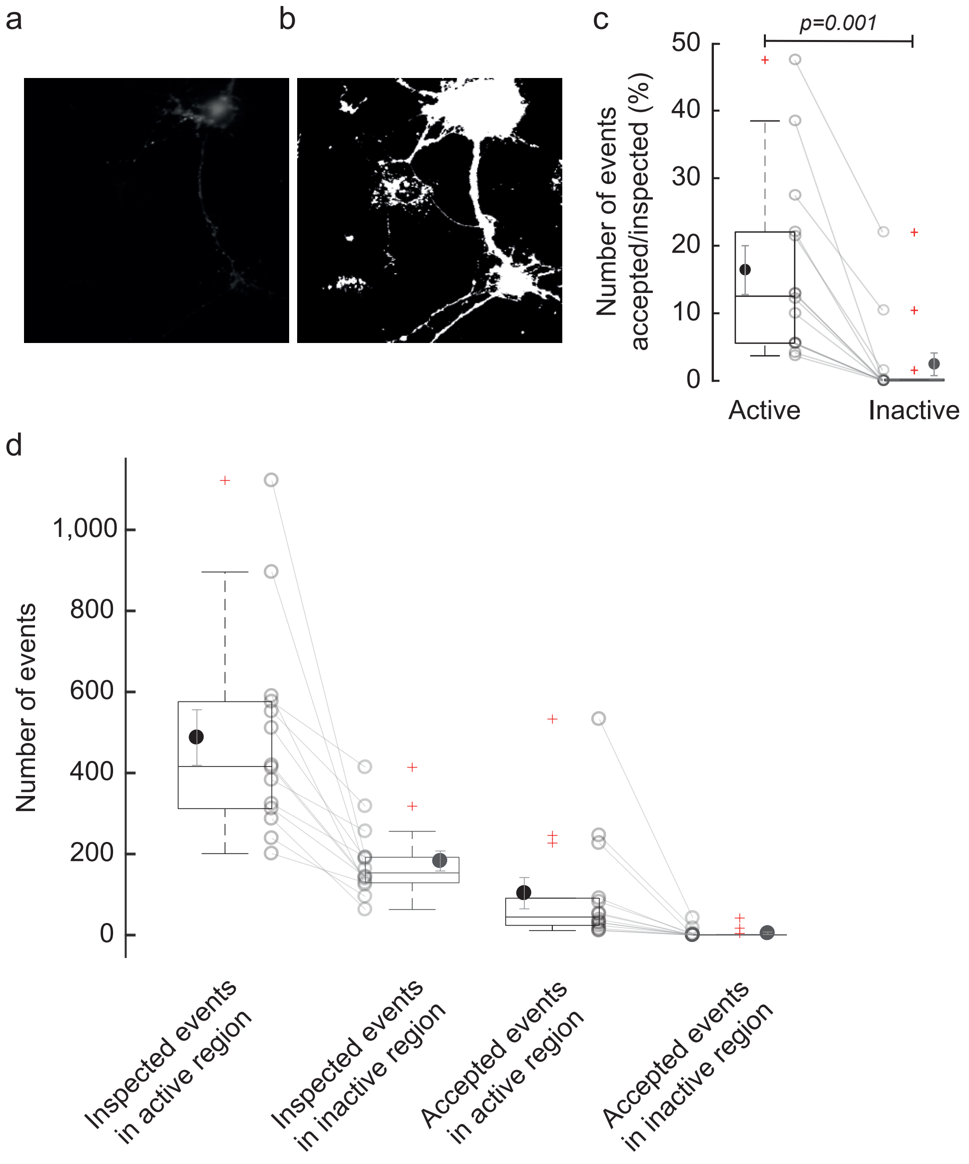

Supplementary Figure 1. Effect of a cell image mask on true positive event detection. To estimate the number of 'true' detections, events were visually inspected ('Inspected events') to mark the events interpreted as biological signals ('Accepted events'). (**a**) Raw 2D image frame. (**b**) 2D image frame representing the neuronal mask calculated by extracting all image regions with above-average pixel intensities. (**c,d**) Automatically detected ROIs representing putative Ca^{2+} rises that were divided in two groups: ROIs that overlapped an inclusion mask corresponding to the neuronal cell structure (Active, black) or not (Inactive, grey). (**c**) The empty dots represent the fraction of accepted over inspected events for each single cell. The statistical analysis was performed with Wilcoxon signed-rank test (n = 14 cells, N = 1 experiment). (**d**) The empty dots represent the number of events per cell. The active and inactive groups are compared for the number of the ROIs which were inspected and the number of accepted ROIs (n = 14 cells, N = 1 experiment).

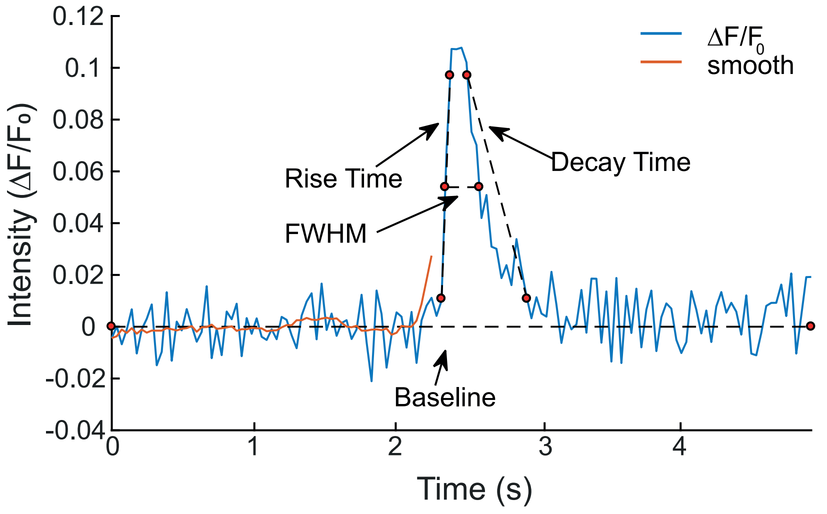

Supplementary Figure 2. Event properties. Trace of an example Ca^{2+} transient and its parameters, which are described in Supplementary Table 3.

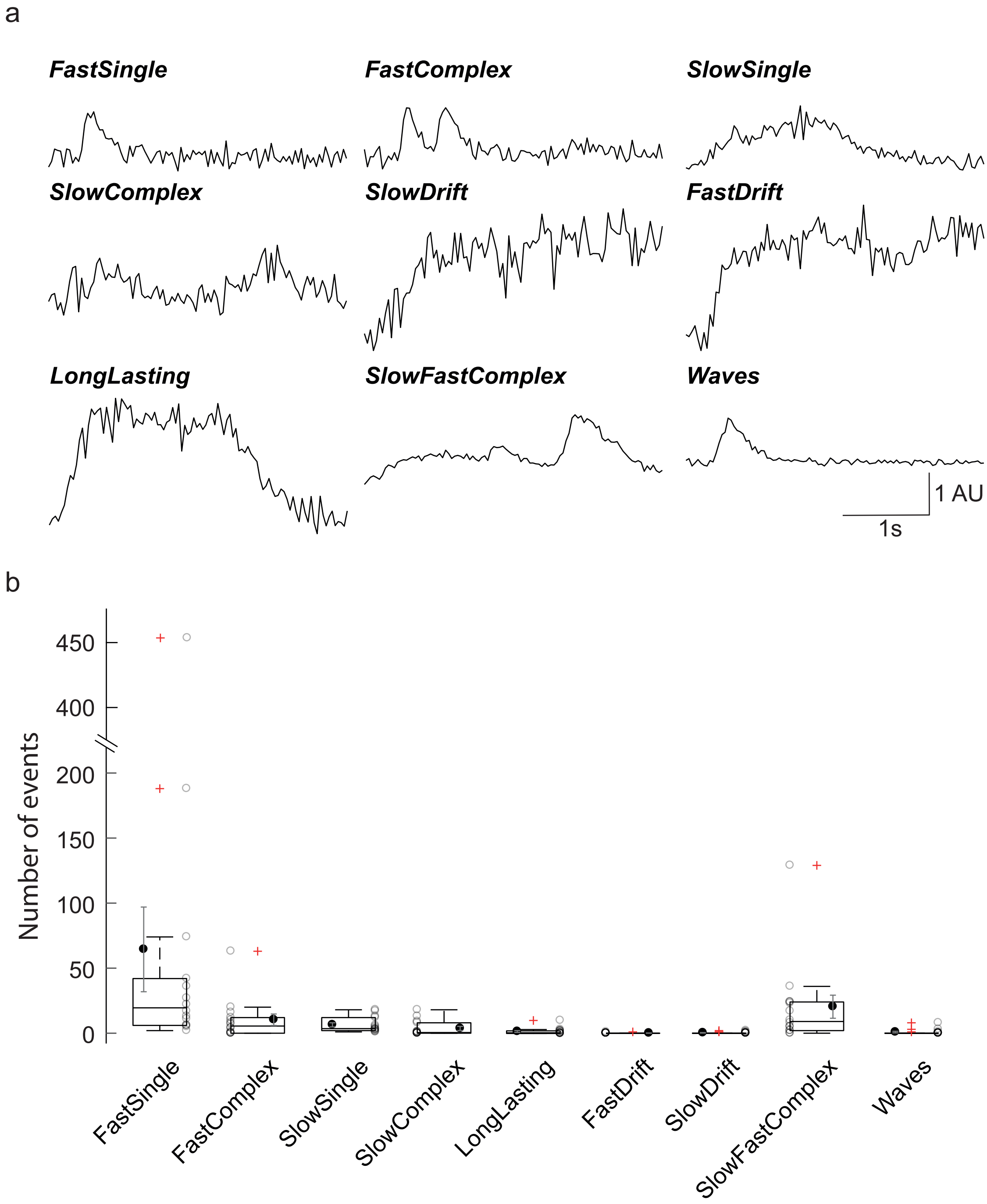

Supplementary Figure 3. Classification of Ca^{2+} events. (**a**) Typical examples of nine different types of Ca^{2+} events, depicted as normalized and scaled traces. In the current version of the event inspection interface, the selected Ca^{2+} events can be assigned to one of nine categories depicted here. Specifications are described in Supplementary Table 2. (**b**) Number of events per Ca^{2+} category, detected in n = 14 cells, N = 1 experiment. The most frequently observed event type was 'FastSingle'. Empty dots represent the averaged parameter per cell.

Supplementary figure 4. Manual vs SICT-assisted SCT parameters.

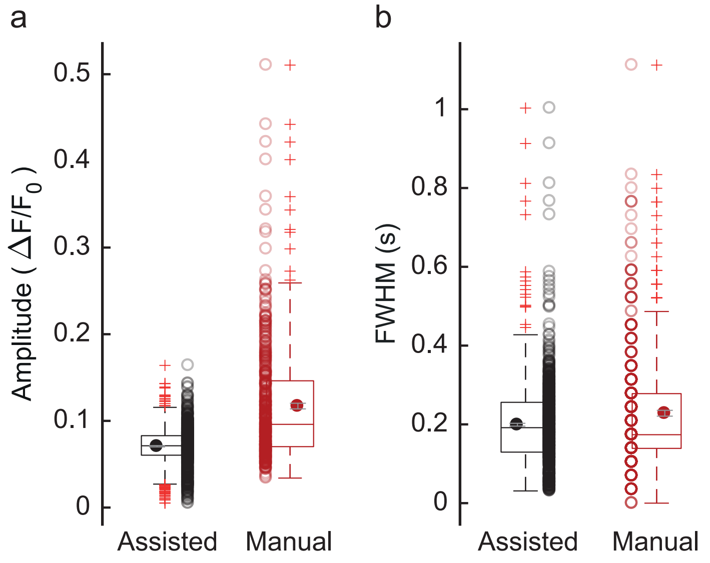

Supplementary Figure 4. Manual versus SICT-assisted SCT parameters. SICT-assisted-processing (Black, n = 850 events), manual-processing (red, n = 414 events from 14 cells, N = 1 experiment). (**a-b**) The box plots show the comparison of the Ca^{2+} transient parameters between the assisted-processing and manual-processing. The empty dots represent the parameter per SCT.

a

b

c

d

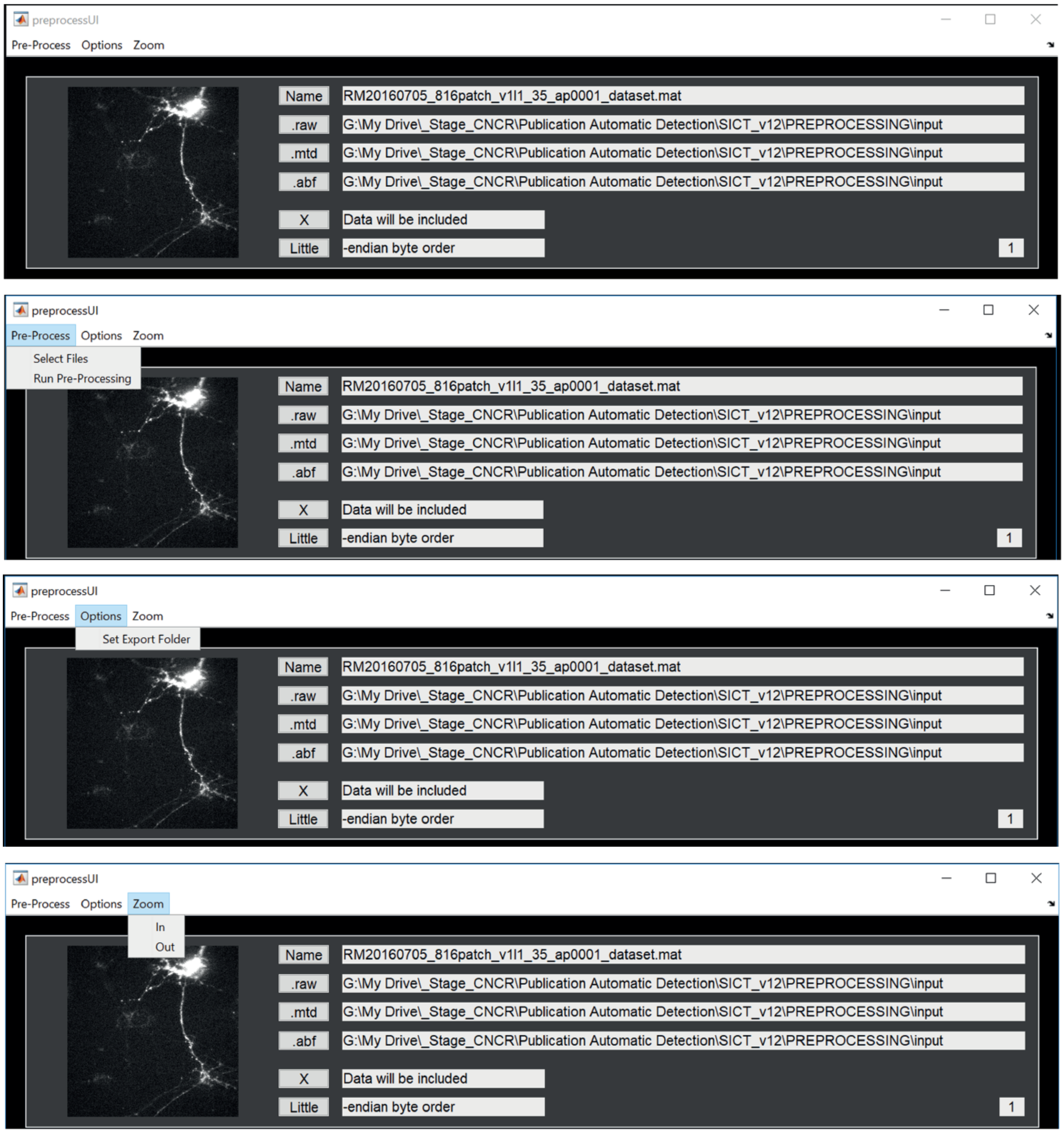

e

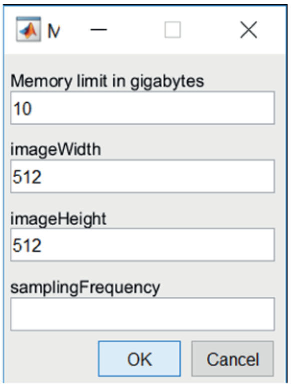

Supplementary Figure 5. Batch processing interface. (**a**) The interface used to process multiple files at once. Each selected '.raw' file, one depicted here, is represented by a tile showing an enhanced contrast preview of the first frame, filenames of any associated '.mtd' or '.abf' file and a name for the output file. The 'X' button will toggle exclusion of the selected '.raw' file from processing and the 'Little' button will toggle between little and big endian byte order, with the preview updating accordingly. (**b**) Selecting Pre-Process > Select Files will open an operating system specific file selection interface so '.raw' files can be selected. Pre-Process > Run Pre-Processing starts the batch processing. (**c**) Option > Select Export Folder allows to choose the location where the file will be saved. (**d**) Zoom > -In or -Out, to in- or decrease the size of the tiles and their text. (**e**) This dialogue window appears when Run Pre-Processing is clicked, to select the memory that will be used by the program and, the pixel specification and the sampling frequency of the images in analysis, before starting the batch processing.

Supplementary Figure 6. Event inspection interface

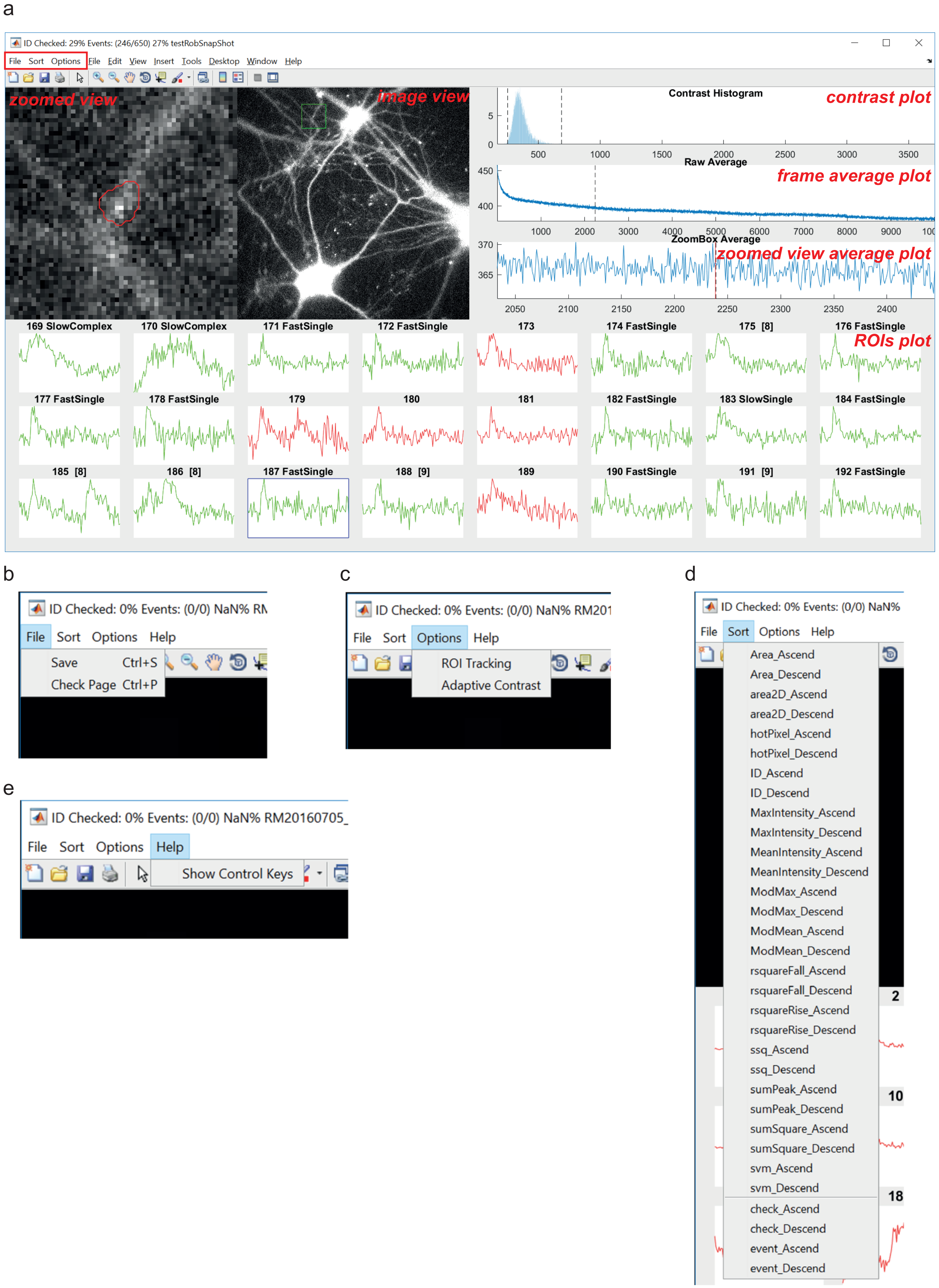

Supplementary Figure 6. Event inspection interface. (**a**) Overview of the user interface for classifying and selecting Ca^{2+} events. The top left shows two images displaying the current frame (right, *image view*) and a zoomed in region (left, *zoomed view*, a region corresponds to the green square in the *image view*). The boundary of a ROI is indicated with a red outline. The top right shows the contrast histogram (top, *contrast plot*), the raw average of each frame over time (middle, *frame average plot), and the zoom box average (*bottom, *zoomed view average plot*), which displays the average intensity of all the pixels inside the green square. The traces on the bottom half of the interface *(ROIs plot*, 24 sets of axes are in a 3 by 8 matrix) display the peak of the average raw intensity of each ROI. In addition, the ID of each ROI is shown together with its user assigned label (if any). All of these graphs are interactive and respond differently to mouse or keyboard input (see text). (**b**) *File > -Save*, to save the session, or -*Check Page*, to mark as inspected all *ROIs plot* in the current view. (**c**) *Option > -ROI tracking*, to show all the detected ROIs in the *image view* when playing the events, or *-Adaptive contrast*, to automatically define the contrast in the *image* and in the *zoomed view* when selecting an event. (**d**) Sorting methods: it sorts the *ROIs plot* base on ROIs features. (**e**) Help function: it will open the supplementary figure 7

Supplementary Figure 7. Control keys for the visual inspection interface

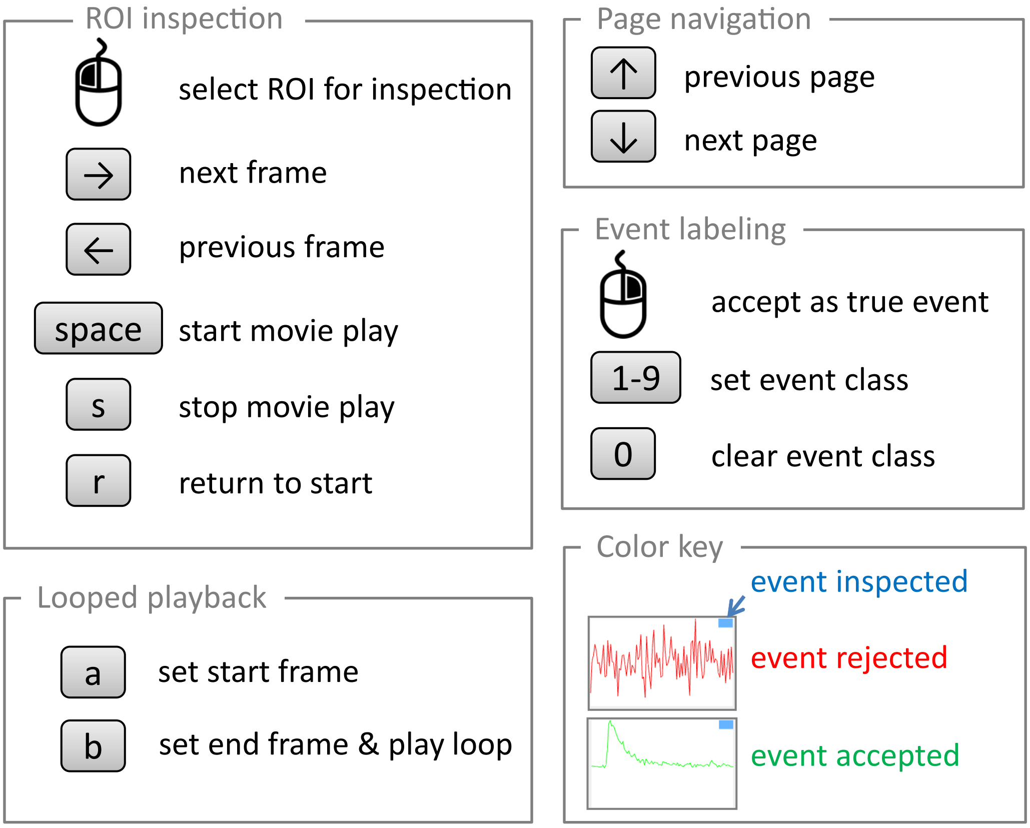

Supplementary Figure 7. Overview of important keyboard / mouse controls for ROI inspection and labelling. This figure is also accessible through the help menu of the graphical user interface.

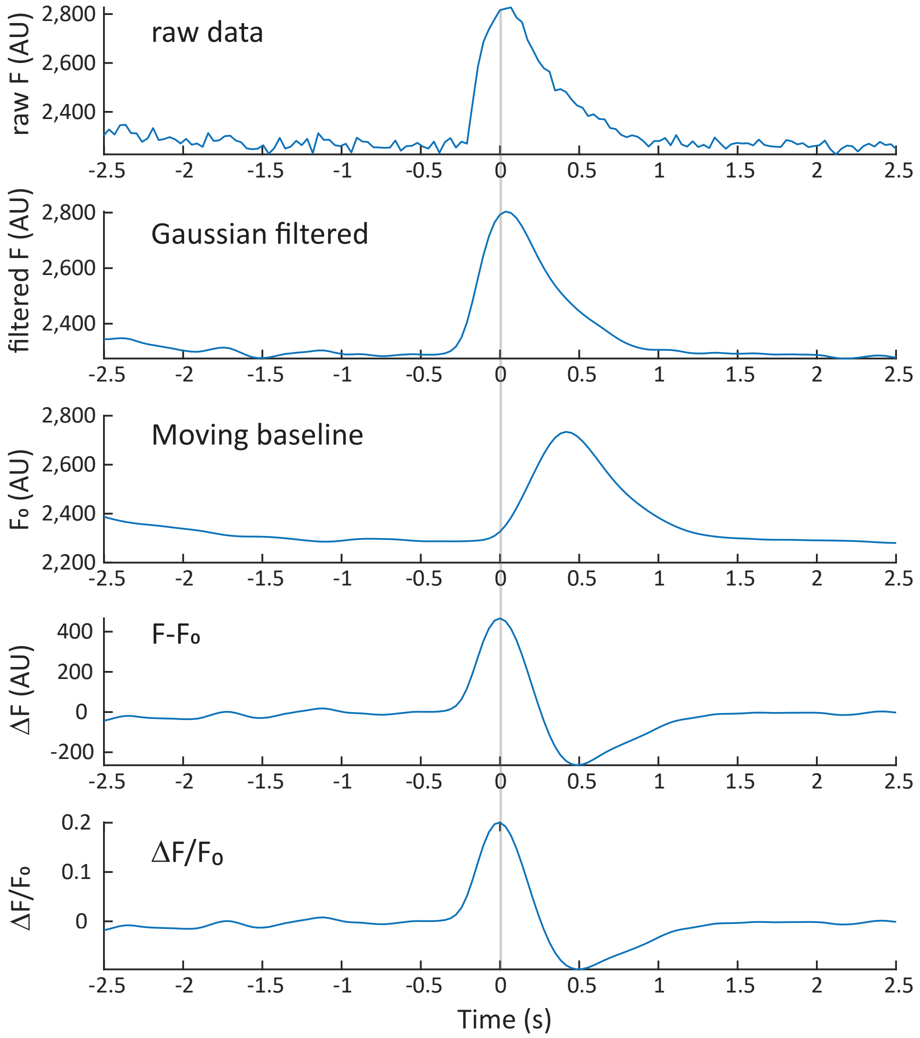

Supplementary Figure 8. Typical example of raw data values and stepwise data processing to calculate $\Delta F/F_0$. To calculate $\Delta F/F_0$ for each pixel, the baseline intensity (F_0) was determined as a moving average of 15 to 5 time points prior to the current data point. The $\Delta F/F_0$ was then calculated as $(F- F_0)/F_0$.

SICT: automatic detection and supervised inspection of fast Ca^{2+} transients.

Supplemental Manual: How to use the interface.

Content:

Content: 11

Introduction 12

Step1-PREPROCESSING: Batch processing interface for ROI detection. 12

Step 2-VISUAL INSPECTION: Manual inspection interface for ROI selection 16

- Interface description 17
- ROIs selection and categorization 18
- Viewing ROI dynamics 19
- Useful functions for ROIs inspection 20
- ROI sorting methods 21
- Saving the results 21

Step 3-POSTPROCESSING: Calculate parameters of accepted events. 21

Introduction

The final versions of the developed scripts are released for MATLAB 2016b, as they make use of functionality that has been introduced in this version. The program is divided in three steps: Preprocessing, Visual inspection and Postprocessing. During each step, be sure to select the corresponding folder as the MATLAB working folder.

Step1-PREPROCESSING: Batch processing interface for ROI detection.

As the first step of the analysis, an interface was created to allow intuitive control over large batches of raw files. The batch pre-processing interface appears by running the command *preprocessUI.m*. Before the interface appears, the user is asked to select the folder that contains the batch of the files to analyse. Once the interface is open, the file list can be modified by selecting the option *Pre-Process>Select Files* in the menu bar **(Supplementary Fig. 5b).** The program searches for '.raw' imaging files in the indicated folder. If imaging files were saved in a format other than '.raw', ImageJ is able to convert various file types to '.raw'. Long filenames exceeding the field size are truncated in the interface. In that case, hovering the cursor over a cropped filename shows the full name in a context menu. For each selected '.raw' file, additional experimental files, '.mtd' and '.abf', are searched in the same folder. The '.mtd' file contains metadata describing the imaging experiment (e.g. image acquisition times). The '.abf' file is the format for electrophysiological recordings (if there are any associated with the imaging data). If those files are found, they're linked to the '.raw' file and saved with the results when the file is processed; however they are not necessary for the pre-processing.

Supplementary Figure 5a shows a screenshot of the interface for one file. Each file is represented by a tile containing the first image frame of the movie. Numbers in the bottom right of each tile indicate their position in the file list. The window is resizable and scales for any size and or aspect ratio. Long file lists can be scrolled through (works poorly with trackpads).

The interface allows for visual inspection of the byte order (big or little), which is a necessity since different camera systems generate different output and incorrect specification leads to detrimental results. Files can be read using 'little' or 'big' endian byte ordering by clicking on '*Little*' or '*Big*' buttons. Files can also be deselected by clicking on the button '*X*' which will change the state 'Data will be included' to 'Data will be skipped' **(Supplementary Fig. 5a)**. These files will not be pre-processed. The option to skip files may be convenient because it avoids the need to reselect all files. Other options in the menu bar include a zoom function to in- or decrease the size of the tiles and their text*: Zoom>in; Zoom>out* **(Supplementary Fig. 5d)**, and the option to set the export folder: *Options> Set Exported Folder* **(Supplementary Fig. 5c)**. .

The pre-processing is started by selecting *Pre-Process> Run Pre-Processing* in the menu bar **(Supplementary Fig. 5b)**. A confirmation dialogue asks for input to set the image dimensions, the sampling frequency of the movie (this value will be used if frame acquisition times cannot be read from the .mtd file) and the maximal amount of RAM to be used by the program **(Supplementary Fig. 5e)**. If the RAM limit does not allow single-pass processing, the movie is processed in separate parts. This requires more computation loops but does not affect detection performance, while preventing RAM overload. After starting is confirmed, process bars appear to indicate which movie file is being processed and how long the batch processing is expected to take.

A note for ImageJ users: please be aware that ImageJ and MATLAB invert x and y coordinates. Therefore, an image of 128 pixels width and 256 pixels height in ImageJ will have an image width of 256 pixels and image height of 128 pixels in MATLAB.

When the pre-processing is completed a message appears. Two output files (denoted '*dataset*' and '*active_mask*' in the filename) are produced for each image file. These files contain definitions of the detected events, called ROIs, saved as a structure array named '*roiStats*' in MATLAB file format ('*.mat' file). This structure array contains many ROI features, specified in **Supplementary Table 1**. The file denoted 'active' contains only ROIs that occur within the neuronal cell mask, as specified in **Supplementary Figure 1** and explained further in the main text. For most downstream applications, this output file can be used. In our experiments, 98.4% of the ROIs interpreted by visual inspection as 'likely biological events' occurred within the mask. The file denoted 'dataset' contains all ROIs regardless of their overlap with the neuronal cell mask.

During preprocessing, a backup copy of the output file is saved in *roiProcessBackups* folder. If a new preprocess needs to be done on the same files, the previous output files need to be deleted from the output and *roiProcessBackups* folder.

Supplementary Table 1. The structure array '*roiStats*' stores the list of detected events (ROIs) and their properties. The field names for each ROI are listed with a description of the property stored in each field. For example, the x, y and t dimension (where it is expressed as frame number) of pixels that are included in the ROI are contained in the field '*roiStats.PixelList'*.

**ROI FEATURES**	**SPECIFICATION**
Area	Total number of pixels (in space and time) of a ROI.
BoundingBox	Coordinates of the smallest cuboid (3D box) that fits the complete ROI. The BoundingBox is defined by six pixel coordinates [x_start, y_start, t_start, x_size, y_size, t_size]. The t coordinates refer to frame numbers, not actual time.
PixelList	List of all pixels that belong to a ROI. For a ROI containing *n* pixels, the PixelList is a *n* by 3 matrix, where the columns indicate the x, y, and t coordinate of each pixel. The t coordinates refer to frame numbers, not actual time.
PixelValues	The raw intensity for each pixel. For a ROI containing *n* pixels, PixelValues is a vertical matrix of *n* by 1 dimensions.
WeightedCentroid	The x, y and t coordinates for the central point of the ROI, contained in a 1 by 3 horizontal matrix.
MeanIntensity	Mean intensity value of all pixels of a ROI.

MaxIntensity	Maximum intensity value of all pixels of a ROI.
uniquePixelList	Unique set of pixels (x, y) that belong to a ROI. For a ROI with an area2D of *n* pixels, the uniquePixelList is a matrix of *n* by 2 elements in which the first column contains the x, and the second the y coordinate of each pixel.
area2D	Number of unique set of pixels that belong a ROI. Pixels included at any one time (image frame) in the 3D Area are included in the 2D area.
meanActivity	The mean raw pixel intensity for the ROI during 100 image frames, starting 15 frames before the peak and 84 frames after, stored in a matrix of 1 x 100 elements.
meanActivityIndex	The frame numbers corresponding to the intensity values in meanActivity, stored in a matrix of 1 by 100 elements.
ModMax	Maximum value of the modulated signal (filtered and expressed as $\Delta F/F_0$) in the ROI. In other words, the peak value of the modulated signal in the x, y and t dimension. (This property is similar to MaxIntensity, except that it uses modulated signal instead of raw signal).
ModMean	Mean value of all pixels in a ROI, calculated from the modulated signal. (This property is similar to MeanIntensity, except that it uses modulated signal instead of raw signal).
ModWeightedCentroid	Similar to WeightedCentroid, except that this property is calculated from the modulated signal
ModPixelvalues	Similar to PixelValues, except that this property is calculated from the modulated signal.
ID	Unique identifier number for the ROI
hotPixel	A measure for the likelihood that a ROI results from hot pixel noise (a type of digital camera noise that is typically active only during a single frame and has an unrealistically high intensity).
ssq	Sum of squares. A measure for the goodness of fit when the meanActivity is fitted with a template waveform. (Smaller value indicates better fit). The template waveform used in our study was an average of many SCTs observed in our instrument. For different instruments, experimental conditions or frame acquisition rates, it is advised to calculate the template waveform empirically[*)].
sumPeak	The sum of 7 data points from normActivity, starting 3 points before the peak and ending 3 points after the peak.
normActivity	Like meanActivity, normActivity contains the mean pixel intensity of the ROI during 100 image frames, starting 15 frames before the peak and 84 frames after, stored in a matrix of 1 x 100 elements. However, normActivity is a normalized measure in which the baseline fluctuates around 0 and the peak value equals 1.
svm	Support vector machine classification based on the data from the human observer comparison (**Fig. 3**).
event	A flag used to indicate if a ROI is thought to represent a true event based on visual inspection. The default value is 0. When

	an event is accepted by right clicking in the manual inspection interface (*step 2*), the value is set to 1.
check	A flag used to indicate if a ROI has been inspected in the manual inspection interface. The default value is 0. When an event is visualized (either by left clicking or after typing CTRL+P in the assisted inspection processing; see *step 2*), the value is set to 1.
eventCategory	The field is empty by default: '[ ]'. When an event is classified in the assisted inspection interface (*step 2*) by pressing a number key (from 1 to 9), the field gets the label of the desired classification (shown in the **Supplementary Table 2**, i.e. 'FastSingle' or ' [8]')

***)** A different template waveform can be specified in the file roiProcess.m, line 49. The template waveform used in our study was:

```
TEMPLATE = [-0.0558052206509354 -0.0492788070873037 -0.0638438041063746 -0.0675059951253681 -0.0663969541949081 -
0.0697563622061362 -0.0604372771256381 -0.0580525337244208 -0.0540587647718514 -0.0773010690552495 -0.0593024952766504
0.282724069787557 0.887593771657547 1 0.975862435295947 0.903047484795952 0.841046337798074 0.768022842229330
0.695654213079397 0.629380946179448 0.557979564764697 0.496583983782349 0.448880826309662 0.395256385002119
0.367154718844817 0.314771077067656 0.297924736608233 0.260141431077273 0.226755023460230 0.213199593991546
0.192091168225410 0.163640472664344 0.147315494876794 0.147643146229577 0.121844637850363 0.114241032289365
0.107667499756225 0.102197336425734 0.0809021799285843 0.0843191777914337 0.0662538521393648 0.0596715938711309
0.0644410806735657 0.0583849842315060 0.0634603080489889 0.0445149920126804 0.0380099565000291 0.0262319592700137
0.0237076041072908 0.0147030817769349 -0.00122574763734354 0.0104645559549760 0.00403456176413428 0.0117747250793551
0.0223917632551007 0.0138278905469966 -9.81645198070868E-06 -0.00359085813459938 0.00959372759258500 0.00496559769867477
0.00843538625884341 -0.0133296510731234 0.00277412932979156 -0.00303764652963347 -0.00903964341410958 -0.0120369334189327 -
0.00885204010958639 -0.00164196520133208 -0.0154883979354039 -0.00415192290114992 -0.0113301488763106 -0.00972810391303416
9.81645198102374E-06 -0.00556461941288452 -0.00476883237230269 -0.00478192097494374 0.000506310778835193 -0.00410174992435886
-0.0102516480186799 -0.00747948197928477 -0.0108537237401730 -0.000742341913130421 -0.00915002396305010 -0.00676746199560598 -
0.0143571063804539 -0.00171046222182092 -0.0279387130544195 -0.0166052920274485 -0.0379440772000688 -0.0338835563740294 -
0.0262590090488053 -0.0199245616572440 -0.0174281288468220 -0.0264714806983466 -0.0365148017916551 -0.0251085208766483 -
0.0169800623497401 -0.0259553534675307 -0.0211806312240395 -0.0235099662074093];
```

Step 2-VISUAL INSPECTION: Manual inspection interface for ROI selection

The manual inspection interface is designed to allow visual inspection of ROIs identified by the detection algorithm **(Supplementary Fig. 6)**. Before starting the inspection, we recommend copying the output file from preprocessing from 'PREPROCESSING\output files' to 'VISUAL INSPECTION\input files'. The inspection interface is opened by calling the function *'viewRoiAlert'* in the folder VISUAL INSPECTION. Optionally, a string value can be passed to specify the input file. For example: viewRoiAlert('input files\filename.mat'). If no filename is specified, the user will be prompted to specify the input file.

Interface description

The manual selection interface is shown in **Supplementary Figure 6a**. In the top left are two image axes (*image view* and *zoomed view*) with the x-pixel dimension on the horizontal axes and the y-dimension on the vertical axes. The *image view* displays the current frame of the raw image stack. The *zoomed view* displays a selected region that corresponds to the green square in the *image view*. Additionally, the *zoomed view* contains a red outline of the ROI that is currently selected. Both images are responsive to mouse input. Clicking inside the axes moves the centre of the green square to the selected position and scrolling while one of the image axes are selected increases or decreases the size of the green square.

In the top right are three plots (*contrast plot*, *frame average plot* and *zoomed view average plot*) that help the user classify the current ROI. The *contrast plot* is a histogram which displays the distribution of values in the raw data file with raw pixel intensity bins on the horizontal axis and bin counts on the vertical axis. For large files, this distribution is based on 100 million random values of the raw data to avoid memory overload. These axes contain two vertical dashed lines which indicate the lower (left) and upper (right) contrast boundaries of the *image* and *zoomed view*. Left clicking will set the lower boundary, right clicking the upper boundary, and double clicking will reset both boundaries to encompass all data points.

The *frame average plot* shows the average intensity for each frame of the raw data file with image frame on the horizontal axis, and average raw frame intensity on the vertical axis. A vertical dashed line indicates the current frame of the image stack. These axes react to a mouse click by jumping to the selected image frame.

The *zoomed view average plot* shows the average trace over time of all pixels in the *zoomed view*, which is depicted also by the green square in the *image view*. Note that this includes both pixels inside and outside of the ROI, which is specified by the

red outline in the *zoomed view*. Just as for the *frame average plot*, the horizontal axis shows imaging frames and the vertical axis shows average raw intensity. Also, the vertical dashed line indicates the current imaging frame. When a ROI is selected, a vertical red line shows the time point of the detected peak. Again, a mouse click jumps to the selected imaging frame. Press the left or right arrow key to jump one frame back or forth, respectively. These axes also react to scrolling by in- or decreasing the number of consecutive frames shown ('temporal zooming').

The remaining part of the user interface shows normalized traces for a set of the ROIs detected by the algorithm (*Rois plot*). For each trace, the horizontal axes display 100 imaging frames, starting 15 frames before the peak and ending 84 frames after. Each experiment generates many pages of these ROIs. To flip through pages, the up and down arrow keys can be used.

ROIs selection and categorization

A glossary of important keyboard and mouse controls is shown in **Supplementary Figure 7** (also accessible through the help menu in the GUI **Supplementary Fig. 6e**). When a ROI is selected by a left mouse click in the *ROIs plot* area, a blue rectangle appears over the selected axes (see ROI #187 in **Supplementary Figure 6a**) and the field '*roiStats.check'* (see **Supplementary Table 1**) is set to 1 (true) to indicate that the ROI was inspected. The imaging frame is reset to 15 frames before the signal peak of the ROI. In the *image view*, the green square is updated to display the location and size of the ROI. In the *zoomed view*, the red outline specifies the pixels that contribute to that ROI. All the *ROIs* that are shown in one page can be altogether labelled as inspected by clicking in the option menu bar *File>Check Page* (or *Ctrl+P*) **(Supplementary Fig. 6b**). <u>For ROIs that are interpreted to likely represent biological events, the inspected ROIs can be accepted as true event by right clicking on the selected ROI.</u> The trace is then plotted in green colour and the ROI data field '*roiStats.event*' is set to 1 (see **Supplementary Table 1**). A second

right click deselects it. If classification of events is wanted, for example, to discriminate events with slow and fast dynamics (**Supplementary Fig. 3a**, main text), labels can be added by pressing number keys according to **Supplementary Table 2** (numpad keys are not recognised). The classification can be helpful because it allows averaging traces for kinetically distinct classes of events.

Supplementary Table 2. Hotkeys for Ca^{2+} event classification. In the manual selection interface, the user can classify Ca^{2+} events by pressing a number key. The '*eventCategory'* field in '*roiStats'* is then set to a string value as specified in the column 'Label'. For our experimental conditions, the following categories are reported as shown in **Supplementary Figure 3a**.

NUMBER KEY	LABEL	SPECIFICATIONS
**1**	**FastSingle**	Representing fast isolated Ca^{2+} transients that have a short duration (<1 s) and contain an obvious peak
**2**	**FastComplex**	For complex peaks that contain more than one peak (this class may also contain 'FastSingle' events that are temporally overlapping
**3**	**SlowSingle**	For events that have a long duration (> 1s) but with intensity still decreasing to baseline eventually
**4**	**SlowComplex**	For slow events with multiple peaks (possibly originating from temporally overlapping single events)
**5**	**Waves**	For events that show directional movement in the spatial dimension (e.g. an event moving along a neurite)
**6**	**Drift**<br>(FastDrift)	For temporary shift in baseline activity, with a fast rise but without a clear decay phase
**7**	**[7] DECIDED BY USER**<br>(SlowDrift)	For temporary shift in baseline activity, with a slow rise but without a clear decay phase
**8**	**[8] DECIDED BY USER**<br>(SlowFastComplex)	For slow and fast event with multiple peak (possibly originating from temporally overlapping 'FastSingle' and 'SlowSingle' events)
**9**	**[9] DECIDED BY USER**<br>(LongLasting)	For temporary shift in baseline activity with a clear rise and decay phase
**0**	**CLEARS THE CURRENT LABEL**	

Viewing ROI dynamics

There is additional functionality to aid the inspection of temporal dynamics of events. When a ROI is selected, the *image view* and *zoomed view* will be set to 15 frames

prior to the peak. Pressing the spacebar will continuously increment imaging frames (like a movie). Pressing the ‘s' key will stop this behaviour. Pressing the ‘r' key will reset the imaging frame to 15 frames prior to the peak (same as when a ROI is selected). With the ‘a' and ‘b' keys, the user can create a time loop. First, the user selects a start frame and presses the ‘a' key, then the user selects an end frame and presses the ‘b’ key. The interface will now increment imaging frames until the ‘b’ frame is reached and then jump back to the ‘a’ frame. Pressing the ‘s’ key stops this behaviour. Note that while ‘playing the raw data video’ most parts of the interface remain interactive. This means that the user can switch active ROIs or change the contrast while imaging frames keep incrementing.

Useful functions for ROIs inspection

The interface offers two functions useful to speed up the ROIs inspection and minimize potential bias. Both *ROI Tracking* and *Adaptive Contrast* can be toggled on and off by accessing the options menu (**Supplementary Fig. 6c**). *ROI Tracking* will display a red outline over the area of each automatically detected ROI in the *image view* during the frames they are active. This feature is useful to check whether the program has recognized all Ca^{2+} transients that are apparent by visual inspection and which of those were selected as true events by the user. As an additional benefit the general activity of the field of view is visualized at a specific time (which will rapidly identify spontaneous action potentials, for example). As a disadvantage, the *ROI tracking* feature demands much computing power and may slow down performance.

Adaptive Contrast, which can similarly be toggled on and off through the options menu, will automatically set the contrast boundaries to the minimum and maximum value of all pixels in a ROI so its dynamics are optimally visible. This function has no computational drawback.

ROI sorting methods

By default, ROIs are sorted by decreasing amplitude of the peak signal. This way of sorting was chosen to cluster together ROIs that are more likely to represent true biological signals, allowing the user to stop manual inspection if the biological signals become smaller than noise. However, ROIs can also be sorted differently if desired (**Supplementary Fig. 6d**). Each sorting method refers to the ROI properties as listed in **Supplementary Table 1**. Useful properties include: 2D area, number of active pixels over the lifetime of the ROI, and support vector machine classification based on the data from the human observer comparison (svm). These sorting methods can assist with finding a visually identified ROI, and get a better impression of the data.

Saving the results

When the inspection process is finished, closing the interface will append the results to '*RoiStats'* of the input file (**Supplementary Table 1**). The user's decisions are stored as Boolean flags ('check=1' for inspected ROIs; 'event=1' for ROIs accepted as true events by the user) and as strings in the '*eventCategory'* field (see **Supplementary Table 2**). It is advised to save regularly to avoid losing work by selecting *File* > *Save*, or by pressing: *Ctrl+S* (**Supplementary Fig. 6b**). This may take a few seconds as the data is written to disk. The results are saved in the folder 'VISUAL INSPECTION\output files'.

Step 3-POSTPROCESSING: Calculate parameters of accepted events.

As the last step, the ROIs flagged to represent true biological events can be processed to study amplitudes, kinetics or timing of those events. Again, we recommend copying the output file from 'VISUAL INSPECTION\output files' to

'POSTPROCESSING\input files'. To start, call the function '*postProcess'* in the POSTPROCESSING folder within the MATLAB environment. Optionally, a string value can be included to limit the analysis to a specific event category as explained in **Supplementary Table 2**. Example: 'postProcess' or postProcess('FastSingle'). The benefit of using a category filter like 'FastSingle' is that it allows plotting the average characteristics for selected event types, which may reflect a distinct biological process. The user is prompted to select a number of input files. Batch processing of multiple files is supported.

The program will only include true events (i.e. when 'event = 1') and ignore all other ROIs. The program is set to extract, for each event, 141 data points, starting from 70 frames prior to the peak and ending 70 frames after. If needed, this number can be changed in line 186 of the code in '*postProcess.m'* ('traceMargin = 70;'). The ROI properties are specified in **Supplementary Table 3** and **Supplementary Figure 2**.

To allow parameter comparisons between experimental groups, the user is asked to enter the number of groups. Filenames can be assigned to the groups in a subsequent dialog. If all measurements belong to a single group, please enter a value of 1 for the number of groups.

Supplementary Table 3. List of the kinetic parameters calculated per single ROI. The table specifies the name of kinetic parameters, which are calculated for each ROI, together with the calculations that are used to extract the parameter itself. The kinetic parameters are stored in the structural array '*eventStats'* together with the ROIs properties specified in **Supplementary Table 1**.

PARAMETER	CALCULATIONS
**PeakIndex**	Frame number indicating the signal peak in the raw data
**Baseline**	Baseline fluorescence intensity just before the rising phase of an event. Calculated by taking a 11-point moving average of the raw intensity (red trace in **Supplementary Figure 2**). The value after the last intensity decrease (dF<0) is defined as the baseline.
**dfTrace**	$\Delta F/F_0$ trace for 141 data points starting 70 frames before the peak and ending 70 frames after. Calculated as $(F_{raw} - F_{Baseline}) / F_{Baseline}$.

**amp**	Amplitude: Maximum value of the $\Delta F/F_0$ trace.
**riseTime**	Rise time: Number of frames in which the intensity increases from 10% to 90% of the peak amplitude, calculated with linear interpolation.
**decayTime**	Decay time: Number of frames in which the intensity decreases from 90% to 10% of the peak amplitude, calculated with linear interpolation.
**FWHM**	Full width at half maximum: Number of frames between the rising and decay phase at 50% the peak amplitude (linear interpolation).
**PeakTime**	If frame acquisition times are known (in our experiments they are read from the metadata), PeakTime is calculated as the time in seconds after the start of image acquisition.

The results are saved in the folder POSTPROCESSING\output files. Two MATLAB (.mat) files are saved: the first has the same title as the input file and contains a structural array '*eventStats*' with all the properties calculated for each extracted Rois per recording (**Supplementary Table 3**). The second (avgPerCell#.mat) contains the averaged features of events per movie and the group number assigned to each movie.

Supplementary table 4. Details of statistical tests.

	**Statistical test (between groups comparison)**	Accepted - significance	**Sample**
figure 3	(1) One-way repeated measures ANOVA	0.01	n = 4 cells, N = 1 independent experiment
	(2) Friedman test		
	(3) One-way repeated measures ANOVA with Greenhouse-Geisser correction		
	**Statistical test (within groups comparison)**	0.05	
	Pairwise comparison		

	**Parameter**	**Statistical result**	**p-value**	**Statistical test**	**Effect size (r)**	**Descriptive and pairwise comparison** *groups*	**Descriptive and pairwise comparison** *comparisons*	***corrected p-value***
figure 3	Frequency	F(3, 9) = 9.594	p = 0.004	(1)	0.87	*orange:* 0.343 ± 0.108 Hz	orange vs yellow	p = 1
						yellow: 0.280 ± 0.096 Hz	orange vs blue	p = 0.165
						blue: 0.171 ± 0.092 Hz	**orange vs purple**	p = 0.014
						purple: 0.179 ± 0.096 Hz	yellow vs blue	p = 0.260
							yellow vs purple	p = 0.635
							blue vs purple	p = 1
	Amplitude	$\chi 2$ (3) = 10.2	p = 0.017	(2)	0.85	orange: 0.074 ± 0.002 $\Delta F/F_0$	orange vs yellow	p = 1
						yellow: 0.072 ± 0.003 $\Delta F/F_0$	orange vs blue	p = 0.171
						blue: 0.088 ± 0.006 $\Delta F/F_0$	orange vs purple	p = 0.602
						purple: 0.085 ± 0.006 $\Delta F/F_0$	**yellow vs blue**	p = 0.037
							yellow vs purple	p = 0.171
							blue vs purple	p = 1
	FWHM	F(1.241, 3.724) = 4.577	p = 0.103	(3)	n.a.			
	Rise time	$\chi 2$(3) = 2.700	p = 0.440	(2)	n.a			
	Decay time	F(3, 9) = 3.433	p = 0.066	(1)	n.a.			

figure 4

Statistical test	Accepted - significance	Sample
(1) Paired samples t-test	0.0167	n = 14 cells, N = 1 independent experiment
(2) Wilcoxon signed-rank test		

Parameter	Statistical result	p-value	Statistical test	Effect size (r)	Descriptive	
					Assisted	*Manual*
Amplitude	t(13) = -8.299	$p < 0.001$	(1)	0.91	0.064 ± 0.003 $\Delta F/F_0$	0.112 ± 0.006 $\Delta F/F_0$
FWHM	Z = -2.166	p = 0.030	(2)	0.40	0.278 ± 0.039 s	0.175 ± 0.016 s
Frequency	Z = -2.273	p = 0.023	(2)	0.42	0.174 ± 0.086 Hz	0.091 ± 0.045 Hz

figure 5

Statistical test (between groups comparison)	Accepted - significance	Sample
(1) Wilcoxon signed-ranks test	0.0125	n = 8 events, N = 1 independent experiment
(2) One-way repeated measures ANOVA		
(3) One-way repeated measures ANOVA with Greenhouse-Geisser correction		
**Statistical test (within groups comparison)**	0.05	
Pairwise comparison		

Parameter	Statistical result	p-value	Statistical test	Effect size (r)	Descriptive	
					Assisted	*Manual*
Area	Z = -2.521	p = 0.012	(1)	0.63	35.54 ± 1.6 μm^2	10.8 ± 0.3 μm^2

Parameter	Statistical result	p-value	Statistical test	Effect size (r)	Descriptive and pairwise comparison			*corrected p-value*
					group 1	*group 2*	*group3*	
Amplitude	F(2,14) = 11.048	p = 0.001	(2)	0.78	assisted: 0.089 ± 0.012 $\Delta F/F_0$	manual: 0.188 ± 0.035 $\Delta F/F_0$	combined: 0.162 ± 0.036 $\Delta F/F_0$	1vs2: p = 0.018<br>1vs3: p = 0.068<br>2vs3: p = 0.239
FWHM	F(2,14) = 0.704	p = 0.511	(2)	n.a				
Integrated amplitude	F(1.198, 8.384) = 27.915	$p < 0.001$	(3)	0.9	assisted: 3.21 ± 0.51 $\Delta F/F_0$ x μm^2	manual: 2.08 ± 0.41 $\Delta F/F_0$ x μm^2	combined: 1.81 ± 0.42 $\Delta F/F_0$ x μm^2	1vs2: p = 0.012<br>1vs3: $p < 0.001$<br>2vs3: p = 0.243

figure 6	Statistical test					accepted - significance		Sample
	(1) Paired samples t-test					0.01		n = 22 cells, N = 3 independent experiments
	(2) Wilcoxon signed-rank test							
	Parameter	Statistical result	p-value	Statistical test	Effect size (r)	Descriptive		
						Baseline	***10mM caffeine***	
	Number of SCTs	Z = -2.761	p = 0.006	(2)	0.42	13.81 ± 3.91	22.04 ± 3.58	
	Integrated amplitude	Z = -2.516	p = 0.012	(2)	0.38	1.89 ± 0.21 $\Delta F/F_0$ x μm^2	3.47 ± 0.5 $\Delta F/F_0$ x μm^2	
	FWHM	Z = -2.971	p = 0.003	(2)	0.45	0.18 ± 0.014 s	0.24 ± 0.036 s	
	Rise time	t(21) = -2.241	p = 0.036	(1)	0.44	0.123 ± 0.008 s	0.14 ± 0.008 s	
	Decay time	Z = -1.737	p = 0.082	(2)	n.a	0.29 ± 0.031 s	0.33 ± 0.019 s	

figure S1	Parameter	Statistical result	p-value	Statistical test	Effect size (r)	Descriptive		Sample
						Active	***Inactive***	
	Fraction of accepted over inspected events	Z = -3.296	p = 0.001	Wilcoxon signed-rank test	0.62	16.39 ± 3.62%	2.44 ± 1.67%	n = 14 cells, N = 1 independent experiment
